# Dietary aflatoxin B1 and antimalarial—a lumefantrine/artesunate—therapy perturbs male rat reproductive function via pro-inflammatory and oxidative mechanisms

**DOI:** 10.1038/s41598-023-39455-1

**Published:** 2023-07-27

**Authors:** Solomon E. Owumi, Angel O. Umez, Uche Arunsi, Chioma E. Irozuru

**Affiliations:** 1 Cancer Research and Molecular Biology Laboratories, Department of Biochemistry, Faculty of Basic Medical Sciences, University of Ibadan, Ibadan, 200004 Nigeria; 2 School of Chemistry and Biochemistry, Parker H. Petit Institute for Bioengineering and Bioscience, Georgia Institute of Technology, Atlanta, GA 30332-0400 USA; 3 Department of Chemistry and Biochemistry, Montana State University, Bozeman, MT 59717 USA; 4 ChangeLab-changing Lives, Cancer Research and Molecular Biology Laboratories, Department of Biochemistry, University of Ibadan, Rm NB 302, Ibadan, 200005 Oyo State Nigeria

**Keywords:** Biochemistry, Risk factors

## Abstract

We investigated the impact of Coartem™ (COA) and aflatoxin B1 (AFB_1_) on rats’ hypothalamus, epididymis, and testis. Male rats were randomly grouped (n = 5 rats) and treated: control group (corn oil), AFB_1_ (70 µg/kg), COA (5 mg/kg), COA + AFB_1_ (5 + 0.035 mg/kg) and COA + AFB_1_ (5 + 0.07 mg/kg) for 28 days. Blood samples were collected for serum prolactin, testosterone, follicle-stimulating and luteinising hormones (FSH and LH) assay upon sacrifice. The semen, hypothalamus, epididymis, and testes were harvested for morphological, biochemical, and histopathology determination of oxidative, inflammation stress, genomic integrity, and pathological alterations. Exposure to the COA and AFB_1_ caused the cauda epididymal spermatozoa to display low motility, viability, and volume, with increased abnormalities. Hormonal disruption ensued in animals exposed to COA and AFB_1_ alone or together, exemplified by increased prolactin, and decreased testosterone, FSH and LH levels. Treatment-related reduction in biomarkers of testicular metabolism—acid and alkaline phosphatases, glucose-6-phosphate dehydrogenase, and lactate dehydrogenase—were observed. Also, COA and AFB_1_ treatment caused reductions in antioxidant (Glutathione and total thiols) levels and antioxidant enzyme (Catalase, superoxide dismutase, glutathione peroxidase, and glutathione-S-transferase) activities in the examined organs. At the same time, treatment-related increases in DNA damage (p53), oxidative stress (xanthine oxidase, reactive oxygen and nitrogen species and lipid peroxidation), inflammation (nitric oxide and tumour necrosis factor-alpha), and apoptosis (caspase-9, and -3) were observed. Chronic exposure to COA and AFB1 led to oxidative stress, inflammation, and DNA damage in male rats' hypothalamic-reproductive axis, which might potentiate infertility if not contained.

## Introduction

Sub-Saharan Africa, a region home to about 1 billion people, is endemic to malaria, and the region alone is responsible for 93% of malaria deaths globally^1^. The World Health Organization, therefore, approved artemisinin-based combination therapy (ACT) for the treatment of malaria and Coartem® (COA), a combination of artemether and lumefantrine, is the most effective drug due to its low resistance to malarial parasites compared to other antimalarial drugs^2^. Artemisinin, the COA’s active agent, targets malaria-infected red blood cells and destroys malaria parasites through the respiratory/oxidative burst process, which is characterised by an enormous generation of free radicals^3^. The sub-Sahara African region is also a high producer and consumer of starch-based crops susceptible to aflatoxin contamination^4^. The area, falling within 40° N and 40° S of the equator, is generally characterised by a warm and humid climate which promotes the growth of the toxin-producing fungi, *Aspergillus flavus* and *Aspergillus paraciticus*^5^. It is, therefore, evident that people in sub-Saharan Africa might be at a higher risk of co-exposure to both COA and aflatoxin B1 (AFB1). Our laboratory has divulged that the exposure of animals to AFB1 resulted in a marked increase in ROS and RNS, thereby perturbing redox signalling along the hypothalamus-pituitary-gonadal axis of rats^6^. Aside from this, experimental animals exposed to AFB1-contaminated feed manifested reduced feed intake, poor feed conversion, feed rejection, decreased body weight, reduced reproductive capabilities, and increased disease incidence due to immune suppression^7, 8^.

Biotransformation of COA is associated with the influx of free radicals in the host. The generation of free radicals is an essential mechanism for the anti-malarial action of COA against *Plasmodium* species. During malarial infection, *Plasmodium* species digest the haemoglobin present in the blood, releasing globin and heme. The globin is proteolysed into amino acids, which are incorporated into the protein pool of the parasites or may be used for energy generation^9^. The reactive free heme from haemoglobin degradation is polymerised into an insoluble crystal called hemozoin^10, 11^. This process is essential for the lifecycle of *Plasmodium* species and serves as an important target of some antimalarial drugs^12, 13^. Alkylation of heme by activated endoperoxides present in artemether prevents its polymerisation to hemozoin, thereby increasing the production of ROS in the parasites and leading to death^14^. This invariably results in elevated ROS production in the host and can promote inflammation and cause extensive damage to host cells and tissues^15^.

Artemether-lumefantrine combination therapy and AFB_1_ have been associated with a significant decrease in testicular and epididymal absolute and relative weights and reductions in sperm volume and motility. Histological examinations revealed a disruption of the seminiferous tubules of the testes and severe vacuolar and necrotic degenerations in the epididymis^6, 16^. In addition, the dysregulation of reproductive hormones by both COA and AFB_1_ is well-reported. These endocrine disruptors have been shown to impair steroidogenesis and alter the serum levels of reproductive hormones by perturbing the redox signalling in the host organisms^6, 17–19^. Recently, our laboratory reported that COA and AFB1 co-exposure is associated with hepatic and renal toxicities, and this is through the induction of oxidative stress, sustenance of pro-inflammatory response, orchestration of apoptosis, and impairment of histological apparatus in the liver and kidney of rats^20^. Taken together, oxidative stress and inflammation are crucial mechanisms of COA and AFB1 toxicities, and current evidence links oxidative stress and inflammation to reproductive abnormality^21^.

In Sub-Saharan Africa, people are constantly exposed to AFB1 through contaminated foods, and when they come down with an ailment, they resort to self-medication without proper diagnosis and treatment. The abuse of antimalarial drugs by these inhabitants and inadvertent exposure to AFB1 can trigger serious health issues via the induction of oxidative stress and sustenance of inflammation in host organisms^20^. Against this backdrop, we hypothesise that co-exposure to COA and AFB1 may impair reproductive functions in rats. To this end, adult male Wistar Albino rats were administered COA and AFB1 (at varying dosages of AFB1) for 28 days. The following endpoints were examined: reproductive hormones, functional testicular enzymes, oxidative stress, inflammatory and apoptotic biomarkers, a marker of genomic instability, apoptosis, and histological scoring of the testis, and epididymis.

## Materials and methods

### Chemicals, reagents, and kits

Coartem (containing 20 mg of Artemether and 120 mg of lumefantrine) was purchased from Novartis® Pharm service AG, Nigeria; aflatoxin B1 (AFB1) (≥ 99%), hydrogen peroxide, reduced glutathione, epinephrine, 1-chloro-2,4-dinitrobenzene (CDNB), thiobarbituric (TBA), 2′,7′-dichlorodihydrofluorescin diacetate, 5′,5′ -dithiobis-2-nitrobenzoic acid (DTNB), *O*-Dianisidine, sodium potassium tartrate, and Sulphosalicylic acid were obtained commercially from Sigma-Aldrich Inc. (St Louis, MO, USA); dipotassium hydrogen phosphate trihydrate, potassium chloride, and potassium dihydrogen phosphate were purchased commercially from AK Scientific, Union City, USA; Folin-Ciocalteu reagent was purchased from J.T Baker, Phillipsburg, PH, USA, follicle stimulating hormone, luteinizing hormone, prolactin, and testosterone were purchased from Fortress Diagnostics, Antrim, UK; trichloroacetic acid (TCA) was purchased from MolyChem, Mumbai India; glucose 6-phosphate dehydrogenase (G6PD), lactate dehydrogenase (LDH), acid phosphatase (ACP), alkaline phosphatase (ALP) were purchased from purchased from Randox™ Laboratories Limited, Crumlin, UK, Luteinizing hormone (BXE0651A), follicle stimulating hormone (BXE0631A), prolactin (PR234F), and testosterone (BXE0862A) were purchased from Fortress Diagnostics, Antrium, UK, and the enzyme-linked immunosorbent Assay (ELISA): Tumor necrosis factor alpha (TNF-α), p53, caspase-9, and caspase-3 were obtained from Elabscience Biotechnology, Wuhan, China. All other reagents and chemicals used were of analytical grade.

### Care of animals, sample size estimation, and experimental outline

**Figure 1:**
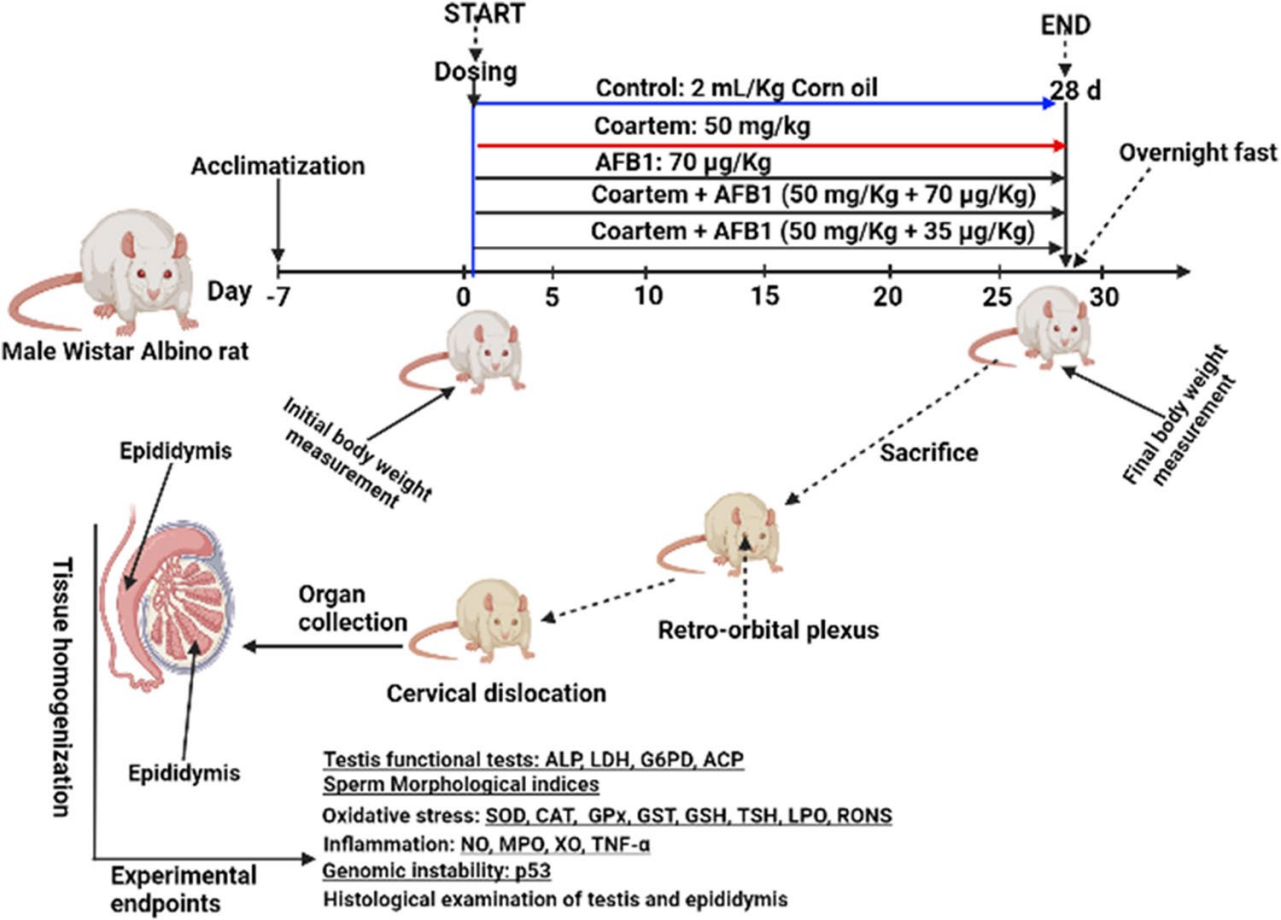
Experimental protocol of experimental rats treated with Coartem (COA: mg/ Kg) and varying doses of aflatoxin B1 (AfB_1_ (D1): mg/Kg) and (AfB_1_ (D2): mg/Kg) for 28 consecutive days. (B): Effect of inadvertent dietary exposure of albino Wistar rats to AfB_1_ and therapeutic doses of COA and experimental endpoints investigated.

This study adopted the 3Rs (replacement, reduction, and refinement) guidelines for the care and use of experimental animals^22–24^. The G* Power software version 3.1.9.4^25^ was used to estimate the sample size at 0.40 effect, 95% power, and 0.05 alpha error of probability for one-way analysis of variance (ANOVA). This yielded a total sample size of 125. The protocol for the design of the in vivo study was sanctioned by the University of Ibadan Animal Care and Use Research Ethics Committee (UI-ACUREC) with an approval number UI-ACUREC/21/0521-7. For this study, forty (40) adult male Wistar rats weighing 130–170 g (aged 7 weeks old) were purchased from the Central Animal House, College of Medicine, University of Ibadan, Nigeria. The animals had unrestricted access to standard rat feed (Breedwell® Feeds Limited, Ibadan, Nigeria) and water. They were maintained under a natural photoperiod of about 12 h’ light/darkness cycle daily and a temperature of 25 ± 2 °C. After the acclimatisation period, which lasted seven days, the animals were randomly grouped into five groups of eight rats. The rats were treated for 28 consecutive days as follows (Fig. 1): Control (corn oil, n = 6), COA alone (5 mg/kg Coartem, n = 6), AFB_1_ alone (70 µg/kg, aflatoxin B_1_, orally administered), COA + AFB_1_1 (5 mg/kg Coartem + 35 µg/kg aflatoxin B_1_, orally administered), COA + AFB_1_2 (5 mg/kg Coartem + 70 µg/kg aflatoxin B_1_, orally administered). COA was administered twice daily on days 26, 27 and 28 of the study. According to doctor’s prescription, COA is usually administered as a total of 6 doses over a period of 3 days. Therefore, rats were exposed to COA twice at days 26, 27 and 28 to mimic human exposure and prevent drug overdose. AFB1 was administered daily for 28 days, and this was in accordance with previous findings that steady exposure to AFB1 could perturb the pituitary-hypothalamic-testicular axis^26^. Separate stock solutions were prepared by dissolving 5.64 g of AFB_1_ in 120 mL of corn oil, and the gavage volume of 0.11 mL and 0.22 mL for Groups IV and V were taken from specific stock solutions. In contrast, COA was prepared by grinding a tablet of Coartem, after which 62.5 mg was weighed using Solid Digital Analytical Balance USS-DBS16 (Cleveland, OH, USA) and dissolved in 25 mL of corn oil. The doses of AFB_1_ and COA used for the current study were derived from previously published works^26, 27^.

### Termination of the experiment and preparation of post-mitochondrial fraction

On precisely the 29th day of experimentation, rats were weighed and sacrificed by cervical dislocation after carbon dioxide (CO_2_) asphyxiation^28^. Blood was collected from the animals in line with the guidelines of the Office of Animal Care and Use^29^. Briefly, a micro haematocrit tube was placed along the medial canthus of the eye, just beside the eyeball and then inserted gently through the conjunctiva. The tube was gently rotated, and blood was collected into sterile non-heparinized sample bottles. After blood collection, gentle pressure was applied to the closed eyelid to prevent further bleeding and aid clotting. The animal was then sacrificed, and the hypothalamus, epididymis and testes were immediately excised, rinsed in 1.15% aqueous potassium chloride (4 °C) and processed for biochemical and histological analyses after the weight was estimated using U.S Solid Digital Analytical Balance USS-DBS16 (Cleveland, OH, USA). The relative organ weight was calculated as shown:

$${\text{Relative}}\;{\text{organ}}\;{\text{weight }}\left( \% \right) \, = \frac{{{\text{Weight}}\;{\text{of}}\;{\text{organ}}}}{Weight \;of\; animal} \times 100$$

The organ was sectioned and fixed in 10% formalin for histological examination. The remaining portion was homogenised in ice-cold potassium phosphate buffer (0.1 M, pH 7.4) with a Teflon homogeniser. The resulting homogenates were centrifuged at 12,000 rpm and 4 °C for 15 min using a cold centrifuge to obtain the post-mitochondria fraction. The supernatant was collected and used for biochemical analyses.

### Determination of experimental rats’ sperm physiognomies and testicular metabolism biomarkers

Experimental rat sperm aberrations and viability were assessed following the established method of Wells and Awa^30^. Epididymal sperm were harvested and released onto a sterile glass slide by dissecting the cauda epididymis. Subsequently, the sperm was diluted by mixing a drop of sperm with sodium citrate dihydrate (2.9%) ten drops. Following that, one drop of diluted sperm was stained with eosin B and a fast green stain freshly prepared by dissolving eosin B (0.2 g) and fast green (0.6 g) in distilled water and ethyl alcohol in a ratio of 2:1. The resulting solution was allowed to incubate for one minute in a water bath at 37 °C. A drop of the dye-stained sperm suspension was finely smeared on a glass slide, allowed to air dry momentarily, and subsequently examined under a Carl Zeiss Axio A10 Light microscope (Jena, Germany) for morphological examination and grading. Spermatozoa (400 hundred from each experimental rat) were enumerated and assigned based on the morphological characteristics used in categorising spermatozoa abnormalities. The viability of the experimental rat sperm was also established and determined by staining with an eosin (1%) and nigrosine (5%) stain reconstituted in sodium citrate dihydrate solution (3%). Spermatozoa motility was determined following the method described by Zemjanis^31^. Accordingly, spermatozoa obtained from the cauda epididymis were diluted with a warm 2.9% sodium citrate dehydrate solution and enclosed with a coverslip. The sperm motility was determined by observing ten microscopic fields at a microscopic magnification of × 200 using a phase-contrast microscope (Leica DM 500, Germany). By scoring the number of progressive sperm, non-progressive and immotile sperm in the same microscopic field of view, the motility of sperm was evaluated, and the data obtained were expressed in terms of percentages of sperm progressive motility. Sperm number was also calculated following the World Health Organisation (WHO) established method^32^, with sperm harvested from the caudal epididymis in saline (0.9%).

The saline-sperm suspension was later filtered through a nylon mesh. After that, a 5 µL aliquot of the filtered sperm suspension was mixed with 95 µL of a diluent containing formalin, NaHCO_3_, and trypan blue in a ratio of 0.35, 5, and 0.25%, respectively. 10 µL of the reconstituted sperm was deposited into the grove of a hemocytometer (Deep 1/10 m; LAB ART, Munich, Germany) and allowed to sediment for 5 min in a humid chamber before counting at (Mag: × 400) under a Carl Zeiss Axio A10 Light microscope (Jena, Germany). Representative biomarkers of testicular metabolism, namely alkaline phosphatase (ALP), acid phosphatase (ACP), glucose-6-phosphate dehydrogenase (G6PD), and lactate dehydrogenase (LDH) activities, were assessed in the supernatant of testicular homogenates using commercially available colourimetric assay kits procured from Randox Laboratories Limited (Antrim, UK). The respective assays were performed following the manufacturers’ specifications.

### Determination of serum levels of reproductive hormones

The sera of the control, COA or AFB_1_ alone and the co-treated rats were assayed for LH, FSH prolactin and testosterone using the respective enzyme-linked immunosorbent assay (ELISA) kits. The data were acquired with a Molecular Devices Spectra Max 340 Microplate Reader using the SoftMax Pro software (version 6.4.0) (Sunnyvale, CA, USA). Inter-assay variation was prevented by testing all of the samples on the same day. The sensitivities of the hormones are prolactin: 0.06 ng/mL; FSH: 0.05 ng/mL, testosterone: 0.08 ng/mL; LH: 0.06 ng.

### Evaluation of hypothalamic, epididymal and testicular antioxidant status and biomarkers of oxidative stress

The supernatants obtained from the hypothalamus, epididymis and testis homogenates were assayed for total protein concentration using the Lowry method^33^. The activities of acid phosphatase (ACP) and alkaline phosphatases (ALP) were measured following the methods of previously reported methods^34,35^; glucose-6-phosphate dehydrogenase (G6PD) was determined in line with the method delineated by Wolf et al.^36^ and lactate dehydrogenase-X (LDH-X) were assessed by the protocol of Vassault^37^.

Antioxidant and oxidative stress biomarkers were then assayed as follows: SOD activity was determined by the method described by Misra and Fridovich^38^; CAT activity was determined using H_2_O_2_ as a substrate according to the process of Clairborne^39^; total sulfhydryl group was determined by the process of Ellman^40^; reduced GSH was determined using the method described by Jollow et al.^41^; GST was assayed using Habig’s method^42^; GPx activity was determined according to the process of Rotruck et al.^43^; lipid peroxidation marker was quantified as malondialdehyde (MDA) according to the method described by Ohkawa and as previously reported^44^ and expressed as μmol MDA/mg protein; and RONS level was determined by the RONS-dependent oxidation of 2′,7′-dichlorodihydrofluorescein diacetate (DCFH-DA) to dichlorofluorescein (DCF)^45^. All readings were measured with a Spectra Max™ 384 multimodal plate reader. Pro-inflammatory biomarkers were assayed as follows: xanthine oxidase –XO– was quantified by Bergmeyer et al. method^46^, nitric oxide –NO– and myeloperoxidase –MPO– levels, were estimated by the protocols of Green et al.^47^ and Granell et al.^48^, respectively. Furthermore, the levels of tumour necrosis factor-alpha (TNF-α), p53, caspase 9 and caspase-3 were estimated as previously reported^49^ using a Spectra Max™ plate reader.

### Epididymal and testicular histopathological assessment

Microscopic assessment of the epididymis and testes sections was performed following Bancroft and Gamble’s methods and reported in the literature^50, 51^. The organs were excised, rinsed, and fixed in Bouin’s solution; after dehydration procedures, the sections were embedded in paraffin and subsequently stained using the haematoxylin and eosin (H&E) stain. All the prepared tissue slides were coded before the slides’ representative images were captured upon examination using a Carl Zeiss Axio light microscope fixed with a Zeiss Axiocam 512 camera (Jena, Germany). The histopathological aberrations and observed lesions in the examined organs were also scored semi-quantitatively following previously reported established methods^52–54^ by a pathologist ignorant of the various treatment cohorts from which the slides were prepared.

### Statistical analysis

The analysis of the data generated from this study was performed by one-way analysis of variance (ANOVA) followed by a post-hoc test (Tukey) using GraphPad Prism version 9.3.1 for Windows (www.graphpad.com; GraphPad, CA, USA). Normality test was performed using the D’Argostino-Pearson Omnibus and Shapiro–wilk tests with a p-value summary indicative of ‘ns” (non-significance). Based on this, the mean difference between the groups was compared using the one-way analysis of variance (ANOVA) followed by a post-hoc Bonferroni test. Bars represent the mean ± SD of 6 rats for the non-ELISA test, while the mean ± SD of 3 rats for ELISA tests (TNF-α, p53, caspase 9 and caspase-3). Groups that differ significantly at *p* < 0.05,* p* < 0.01, *p* < 0.001* p* < 0.0001 are indicated by *, **, *** and ****, respectively; pairwise comparison lines represent compared groups.

### Ethical statement

All experiments were performed according to relevant guidelines and regulations and adhered to the ARRIVE guidelines (https://www.arriveguidelines.org) to report animal experiments. The protocols for the care and use of experimental animals in this study were approved by the University of Ibadan, Animal Care and Use in Research Ethical Committee with approval number: UI-ACUREC/032-0521-7.

## Results

### Exposure to COA and AFB1 alters male rats' mean body weight and organosomatic indices

The effect of exposure to COA and AFB1 on the body weight gain, organ weight and relative organ weight of experimental animals is displayed in Table 1. Compared to the control, the animals treated with AFB_1_ alone had their body weight gain decreased. In contrast, animals treated with COA alone demonstrated a significant (*p* < *0.05*) reduction in weight gain. In addition, the weight and relative weight of the testes, epididymis and hypothalamus of the animals exposed to COA or AFB_1_ only reduced except for the relative testes weight, which increased slightly compared to the control group. In the co-treated groups, the body weight gain, organ weight and relative organ weight of the animals decreased compared to the control except for testicular weight and relative weight, which increased. Our previous in-silico probing supports the observed toxicity using the Protox II and SwissAdme. We documented LD50 of 1000 mg/kg and 3 mg/kg for COA and AFB1, respectively^20^.

**Table 1:** Body weight gain, organ weight and relative organ weight of rats following exposure to AFB1 and COA for 28 days.

	Control	AFB1	COA	COA + AFB1_1_	COA + AFB1_2_
Initial body weight (g)	169.80 ± 15.77	168.30 ± 6.83	133.70 ± 5.89*	160.00 ± 5.44**	172.60 ± 7.23**
Final body weight (g)	232.80 ± 24.11	225.70 ± 9.25	169.70 ± 9.52*	237.20 ± 23.26**	211.00 ± 23.98**
Body weight gain (g)	63.00 ± 25.20	57.33 ± 10.67	36.00 ± 8.25	77.17 ± 19.20	67.17 ± 54.44
Testis weight (g)	2.50 ± 0.17	2.46 ± 0.20	1.91 ± 0.22*	2.71 ± 0.25	2.50 ± 0.16
Epididymis weight (g)	0.37 ± 0.06	0.31 ± 0.04	0.17 ± 0.10*	0.34 ± 0.03	0.30 ± 0.07
Hypothalamus weight (g)	0.06 ± 0.00	0.03 ± 0.01*	0.04 ± 0.00*	0.04 ± 0.00*	0.05 ± 0.00
Relative testis weight (%)	1.08 ± 0.06	1.09 ± 0.12	1.14 ± 0.18	1.15 ± 0.11	1.19 ± 0.16
Relative epididymis weight (%)	0.16 ± 0.03	0.14 ± 0.01	0.10 ± 0.07	0.15 ± 59.26	0.14 ± 0.03
Relative hypothalamus weight (%)	0.03 ± 0.00	0.01 ± 0.01*	0.02 ± 0.00	0.02 ± 0.00	0.02 ± 0.01

AFB1 alone: 0.070 mg/kg; COA alone: 5 mg/kg; AFB1_1_: 0.035 mg/kg; AFB1_2_: 0.07 mg/kg. Data analysis was performed by one-way analysis of variance (ANOVA) followed by a post-hoc test (Tukey). Data were expressed as mean ± SD of 5 rats. *, **: significant when compared to control at p < 0.05 and 0.01 respectively.

### Exposure to COA and AFB1 impairs the reproductive hormones, testicular metabolic enzymes, and sperm functional parameters of rats

**Figure 2:**
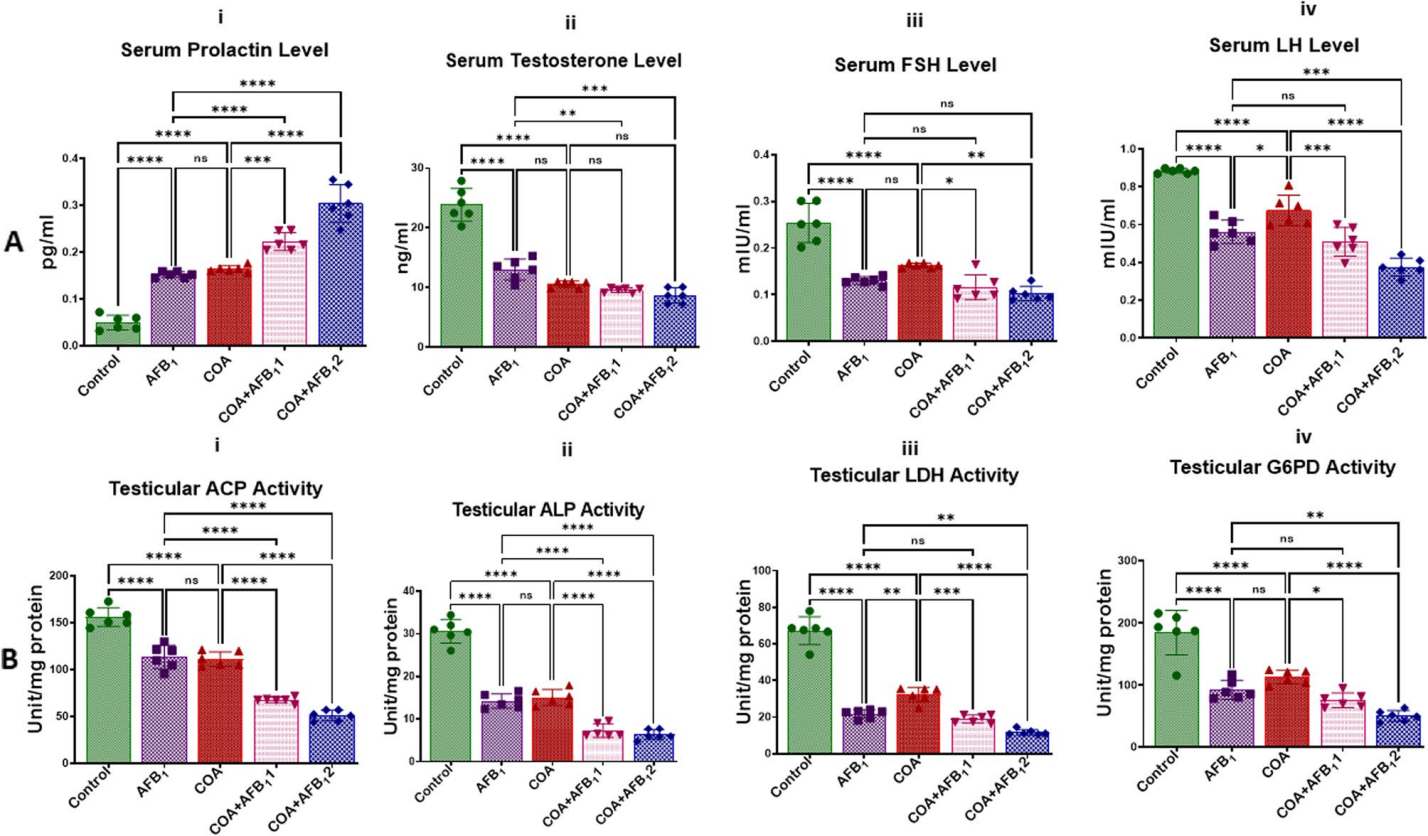
Effect of COA and AFB_1_ exposure on reproductive functions of rats. (**A i–iv**): COA and AFB_1_ alters rat’s serum reproductive hormone levels. (**B i–iv**): COA and AFB_1_ decreased rat’s testicular metabolic enzymes activities. COA, coartem at 5 mg/kg; AFB_1_1, aflatoxin at 35 µg/kg; AFB_1_2, aflatoxin at 70 µg/kg. The mean difference between the groups was compared with a one-way analysis of variance (ANOVA) followed by a post-hoc Bonferroni test. Bars represent the mean ± SD of 6 rats. Groups that differ significantly at *p* < 0.05,* p* < 0.01, *p* < 0.001* p* < 0.0001 are indicated by *, **, *** and ****, respectively; pairwise comparison lines represent compared groups. FSH, follicle-stimulating hormone; LH, luteinising hormone; ACP, acid phosphatase; ALP, alkaline phosphatase; LDH, lactate dehydrogenase; G6PD, glucose-6-phosphate dehydriogenase.

The effects of exposure to COA and AFB1 on the serum levels of reproductive hormones (prolactin, testosterone, FSH and LH), testicular metabolic enzymes (ACP, ALP, LDH, and G6PD) along with spermatogenesis and testicular sperm functional indices of rats are presented in Fig. 2A (i–iv) and B (i–iv) and Table 2. The serum levels of testosterone, LH and FSH were significantly (*p* < *0.05*) decreased in the groups treated with AFB1 or COA alone compared to the control. The reduction in the levels of these hormones is even more pronounced in the groups co-treated with AFB1 (35 and 70 µg/kg) and COA. On the contrary, the serum level of prolactin was significantly elevated when animal cohorts were treated with AFB_1_ or COA alone, and the prolactin level was increased considerably in the cohorts co-exposed to both AFB_1_ (35 and 70 µg/kg) and COA compared to the control (Fig. 2A i–iv). The results further indicate the toxic effects of exposure to COA and AFB_1_ as seen in the significant decreases in the activities of ACP, ALP, LDH and G6PD in the animals treated with COA and AFB1, compared to the control animals (Fig. 2 B i–iv). In addition, we probe the effect of exposure to COA and AFB_1_ on sperm motility, epididymal sperm number, sperm viability, and total sperm abnormality in rats. Compared to the control, the sperm motility, sperm number and sperm viability of rats exposed to COA or AFB1 alone and those in the co-exposed groups showed a noticeable reduction with a concurrent increase in total sperm abnormalities (Table 2). The results divulge that COA and AFB1 can impair reproductive functions.

**Table 2:** Sperm analysis and sperm abnormalities of rats following exposure to COA and AFB_1_ for 28 days.

	Control	AFB_1_ alone	COA alone	COA + AFB_1_1	COA + AFB_1_2
Sperm analysis					
Motility	92.00 ± 2.74	76.00 ± 5.47****	62.00 ± 4.47****	70.00 ± 0.01****	70.00 ± 0.02****
Viability	96.80 ± 1.64	95.20 ± 1.46	95.30 ± 1.64	94.60 ± 1.53	94.20 ± 1.23
Epididymal sperm count	5.18 ± 0.04	5.16 ± 0.05	5.18 ± 0.05	5.18 ± 0.04	5.16 ± 0.05
Sperm abnormality					
Abnormality of the head (%)	2.14 ± 0.23	2.23 ± 0.32	2.38 ± 0.48	2.080 ± 0.55	2.29 ± 0.44
Abnormality of the tail (%)	16.03 ± 1.35	18.37 ± 1.09**	19.71 ± 1.93****	19.80 ± 0.90****	20.30 ± 0.48****
Abnormality of the mid-piece (%)	4.23 ± 0.32	4.76 ± 0.33	5.31 ± 0.33****	5.51 ± 0.18****	5.64 ± 0.43****
Total abnormality (%)	11.45 ± 0.95	12.96 ± 0.9484	13.95 ± 1.28***	13.94 ± 0.76***	14.36 ± 0.38****

COA: 5mg/kg; AFB_1_1: 35 µg/kg; AFB_1_2: 70 µg/kg. Values are expressed as mean + SD of 6 rats. Groups that differ significantly at *p* < 0.05,* p* < 0.01, *p* < 0.001* p* < 0.0001 are indicated by *, **, *** and **** respectively compared to the control.

### Exposure to COA and AFB1 impairs redox homeostasis in rats' hypothalamus, testis, and epididymis

**Figure 3:**
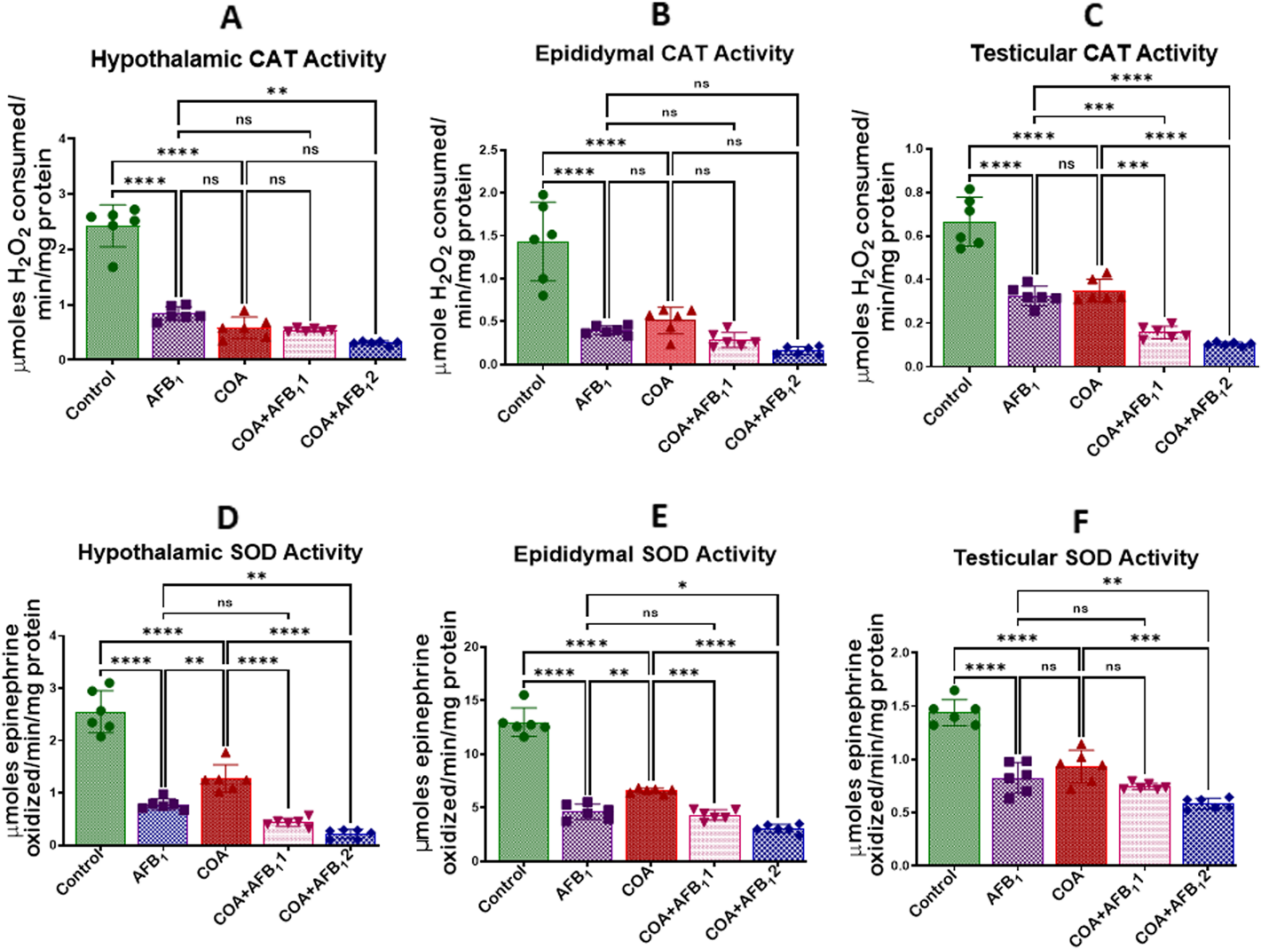
Effects of COA and AFB_1_ exposure on hypothalamic, epididymal and testicular activities of CAT and SOD in male rats. COA, coartem at 5 mg/kg; AFB_1_1, aflatoxin at 35 µg/kg; AFB_1_2, aflatoxin at 70 µg/kg. The mean difference between the groups was compared with a one-way analysis of variance (ANOVA) followed by a post-hoc Bonferroni test. Bars represent the mean ± SD of 6 rats. Groups that differ significantly at *p* < 0.05,* p* < 0.01, *p* < 0.001* p* < 0.0001 are indicated by *, **, *** and ****, respectively; pairwise comparison lines represent compared groups. CAT, catalase; SOD, superoxide dismutase.

**Figure 4:**
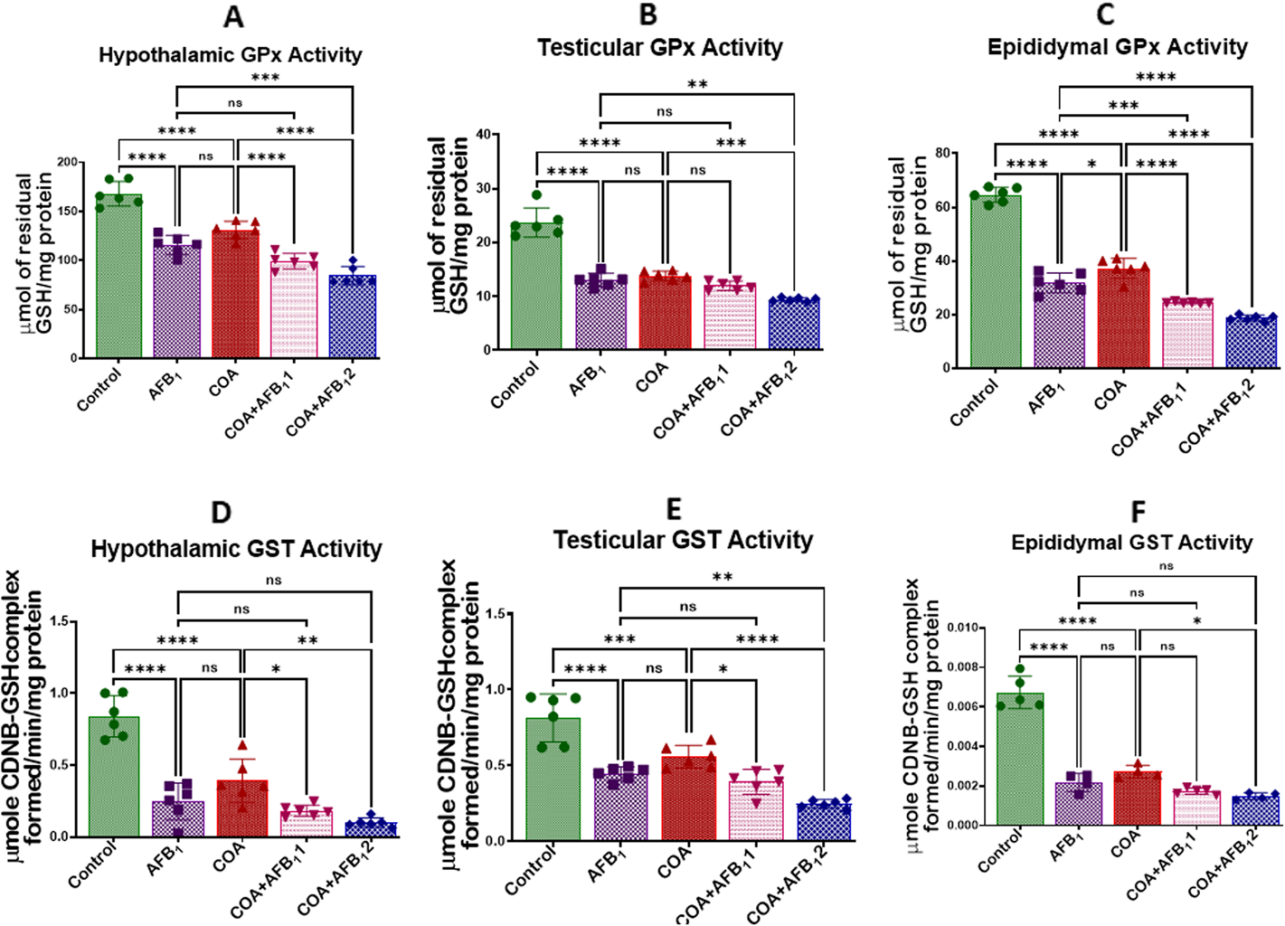
Effect of COA and AFB_1_ exposure on the hypothalamic, epididymal and testicular activities of GPx and GST in male rats. COA, coartem at 5 mg/kg; AFB_1_1, aflatoxin at 35 µg/kg; AFB_1_2, aflatoxin at 70 µg/kg. The mean difference between the groups was compared with a one-way analysis of variance (ANOVA) followed by a post-hoc Bonferroni test. Bars represent the mean ± SD of 6 rats. Groups that differ significantly at *p* < 0.05,* p* < 0.01, *p* < 0.001* p* < 0.0001 are indicated by *, **, *** and ****, respectively; pairwise comparison lines represent compared groups. GPx, glutathione peroxidase; GST, glutathione-*S*-transferase.

**Figure 5:**
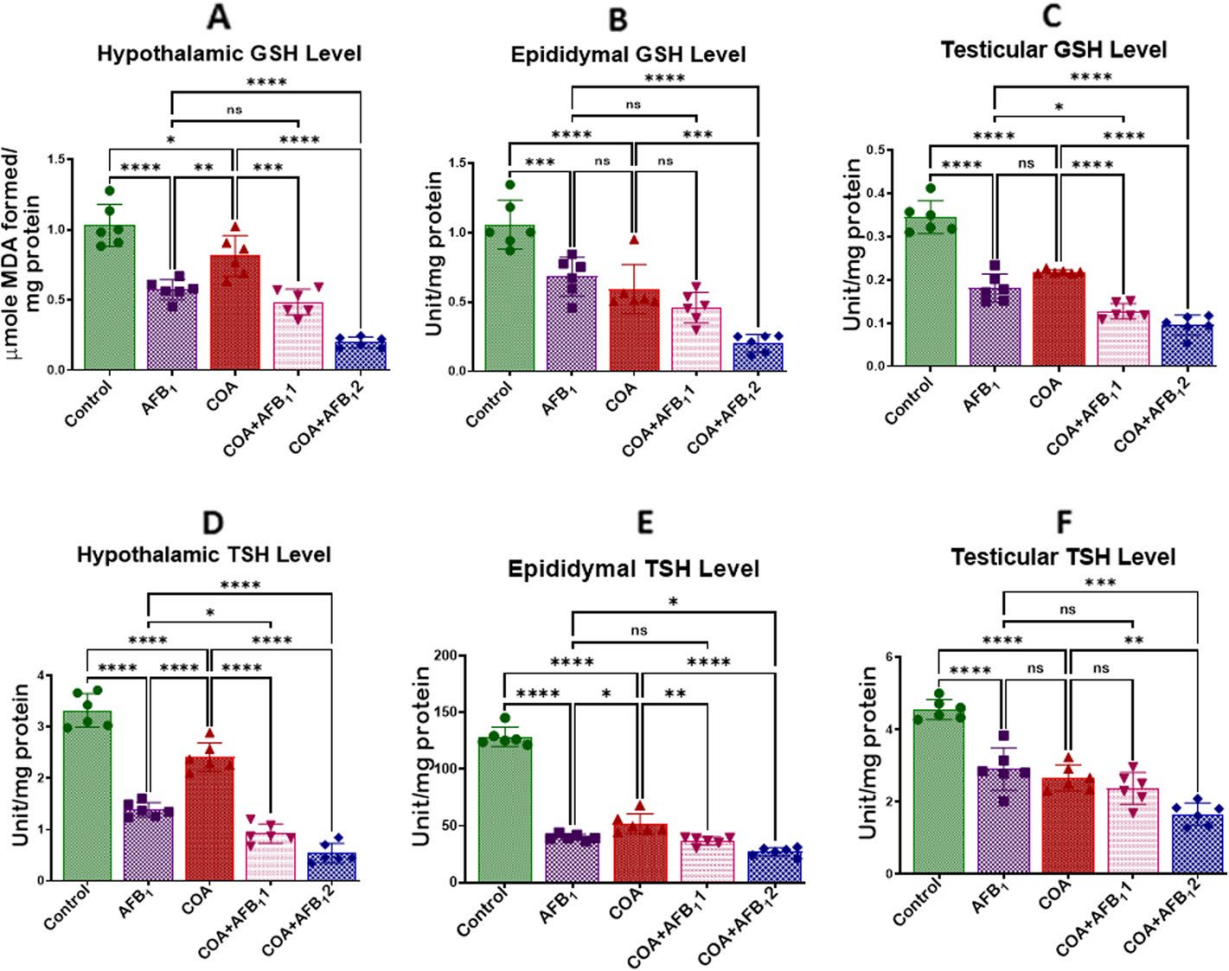
Effect of COA and AFB_1_ exposure on the hypothalamic, epididymal and testicular levels of GSH and TSH in male rats. COA, coartem at 5 mg/kg; AFB_1_1, aflatoxin at 35 µg/kg; AFB_1_2, aflatoxin at 70 µg/kg. The mean difference between the groups was compared with a one-way analysis of variance (ANOVA) followed by a post-hoc Bonferroni test. Bars represent the mean ± SD of 6 rats. Groups that differ significantly at *p* < 0.05,* p* < 0.01, *p* < 0.001* p* < 0.0001 are indicated by *, **, *** and ****, respectively; pairwise comparison lines represent compared groups. GSH, glutathione; TSH, total sulfhydryl group.

The effect of COA and AFB1 on the activities of CAT, SOD, GPx, GST, GSH, and TSH in rats' hypothalamus, epididymis and testis are displayed in Figs. 3, 4 and 5A–F). Compared to the control, the activities of CAT, SOD, GPx, GST, GSH, and TSH of animals treated with COA or AFB_1_ decreased in the hypothalamus, epididymis, and testis. The results show that the administration of AFB1 and COA significantly reduced (*p* < *0.05*) the tissue levels of CAT and SOD in the hypothalamus, epididymis, and testis compared to the control. The results further show that in cohorts of rats treated with 35 and 70 µg/kg at a constant dosage of COA, the tissue level of CAT was significantly decreased (*p* < *0.05*) in the hypothalamus (at 70 µg/kg), and testis (at 35 and 70 µg/kg). In contrast, the level of SOD decreased significantly in the hypothalamus, epididymis, and testis at 35 and 70 µg/kg compared to AFB1-alone treated cohorts (Fig. 3A–F). In addition, exposure to COA and AFB1 significantly decreased (*p* < *0.05*) the tissue levels of GPx and GST compared to the control. As the doses of AFB1 were increased and that of COA was maintained, the tissue level of GPx was significantly decreased in the hypothalamus, epididymis, and testis compared to AFB1, and the level of GST was diminished considerably (*p* < *0.05*) in the epididymis at 35 and 70 µg/kg (Fig. 4A–F). The results further reveal that the administration of COA and AFB1 significantly reduced (*p* < *0.05*) the GSH and TSH tissue levels compared to the control. As the concentration of AFB1 was increased, and that of COA was maintained, the tissue levels of GSH and TSH were significantly reduced in the hypothalamus, epididymis, and testis compared to AFB1 (Fig. 5A–F). This shows that exposure to COA and AFB1 can dampen the expression of endogenous antioxidants in the target tissues.

### Exposure to COA and AFB1 induces oxidative stress in rats’ organs examined

**Figure 6:**
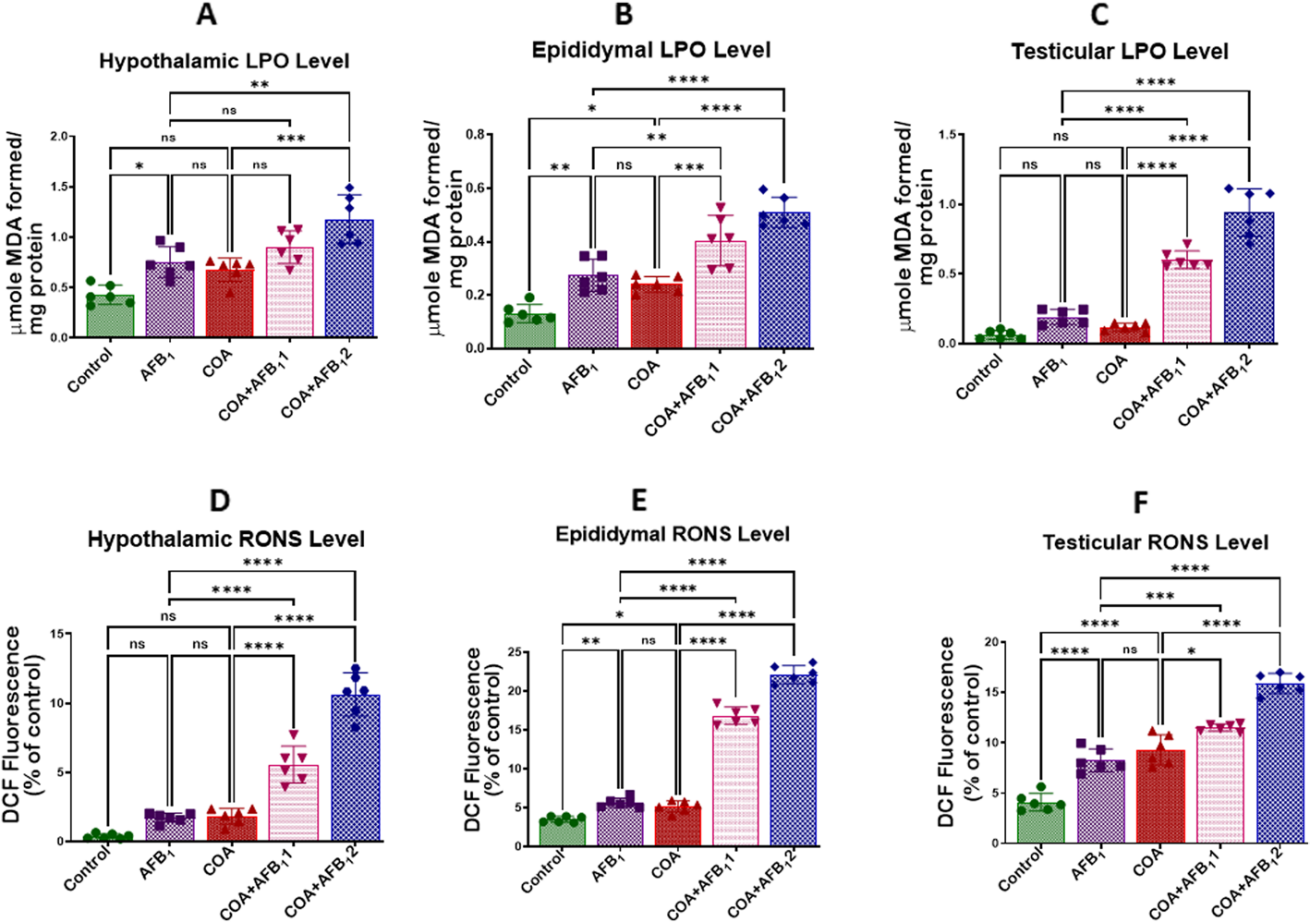
Effect of COA and AFB_1_ exposure on the hypothalamic, epididymal and testicular levels of LPO and RONS in male rats. COA, coartem at 5 mg/kg; AFB_1_1, aflatoxin at 35 µg/kg; AFB_1_2, aflatoxin at 70 µg/kg. The mean difference between the groups was compared with a one-way analysis of variance (ANOVA) followed by a post-hoc Bonferroni test. Bars represent the mean ± SD of 6 rats. Groups that differ significantly at *p* < 0.05,* p* < 0.01, *p* < 0.001* p* < 0.0001 are indicated by *, **, *** and ****, respectively; pairwise comparison lines represent compared groups. LPO, lipid peroxidation; RONS, reactive oxygen and nitrogen species.

The effect of co-exposure to COA and AFB1 on the oxidative stress biomarkers in rats' hypothalamus, epididymis and testes are displayed in Fig. 6A–F. Compared to the control, the levels of LPO & RONS in animals treated with COA or AFB_1_ alone were significantly (*p* < *0.05*) raised in the hypothalamus (Fig. 6A and D), epididymis (Fig. 6B and E), and testis (Fig. 6C and F) compared to the control animals. In addition, as the concentration of AFB1 was increased, and that of COA was maintained, the tissue levels of LPO and RONS were significantly elevated in the hypothalamus, epididymis, and testis compared to the rat cohort treated with AFB1 alone**.** This shows that COA and AFB1 may generate free radicals, triggering oxidative stress in the target tissues.

### Exposure to COA and AFB1 orchestrates pro-inflammatory responses in rats’ hypothalamus, testis, and epididymis

**Figure 7:**
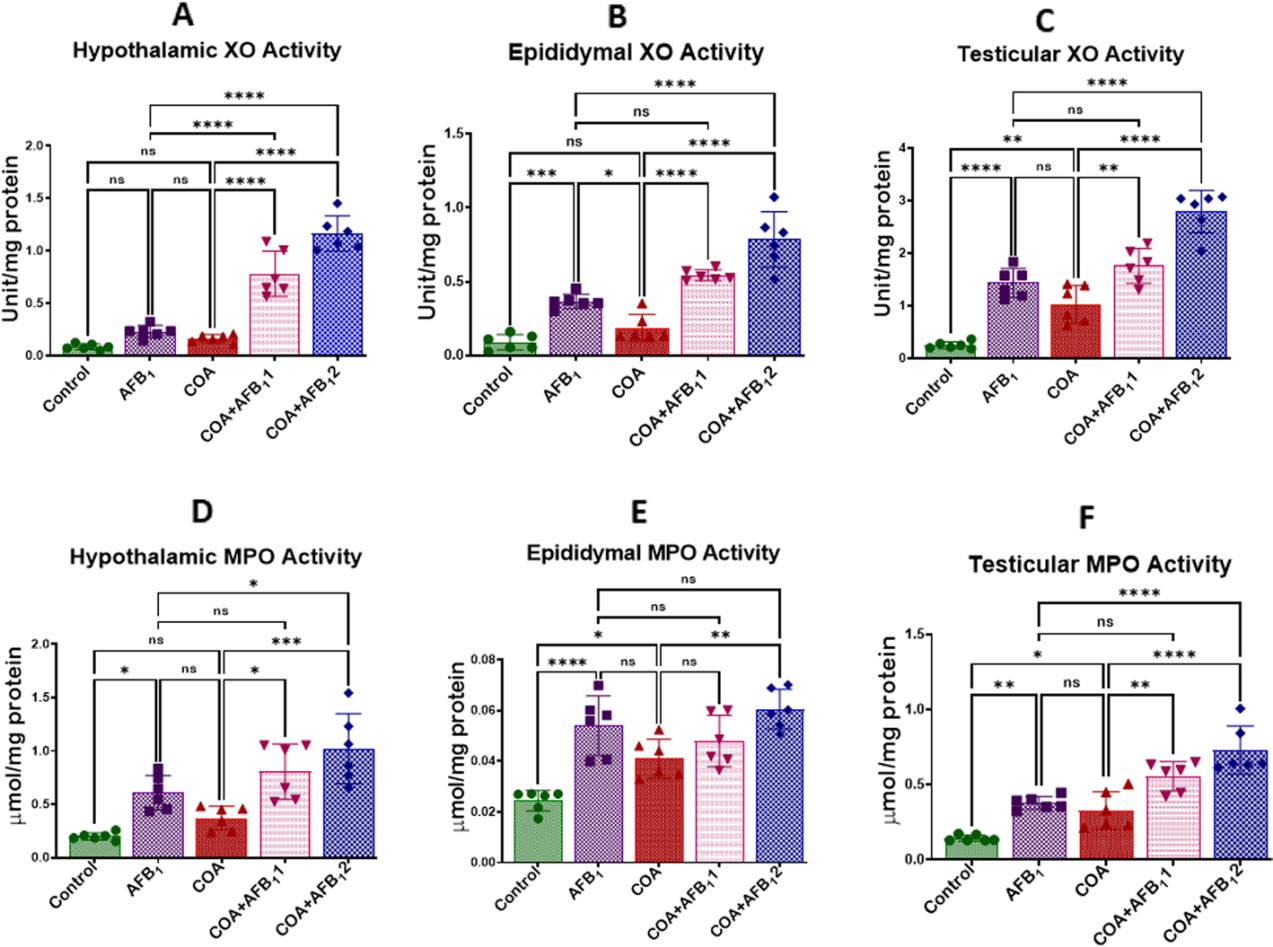
Effect of COA and AFB_1_ exposure on the hypothalamic, epididymal and testicular activities of XO and MPO in male rats. COA, coartem at 5 mg/kg; AFB_1_1, aflatoxin at 35 µg/kg; AFB_1_2, aflatoxin at 70 µg/kg. The mean difference between the groups was compared with a one-way analysis of variance (ANOVA) followed by a post-hoc Bonferroni test. Bars represent the mean ± SD of 6 rats. Groups that differ significantly at *p* < 0.05,* p* < 0.01, *p* < 0.001* p* < 0.0001 are indicated by *, **, *** and ****, respectively; pairwise comparison lines represent compared groups. XO, xanthine oxidase; MPO, myeloperoxidase.

**Figure 8:**
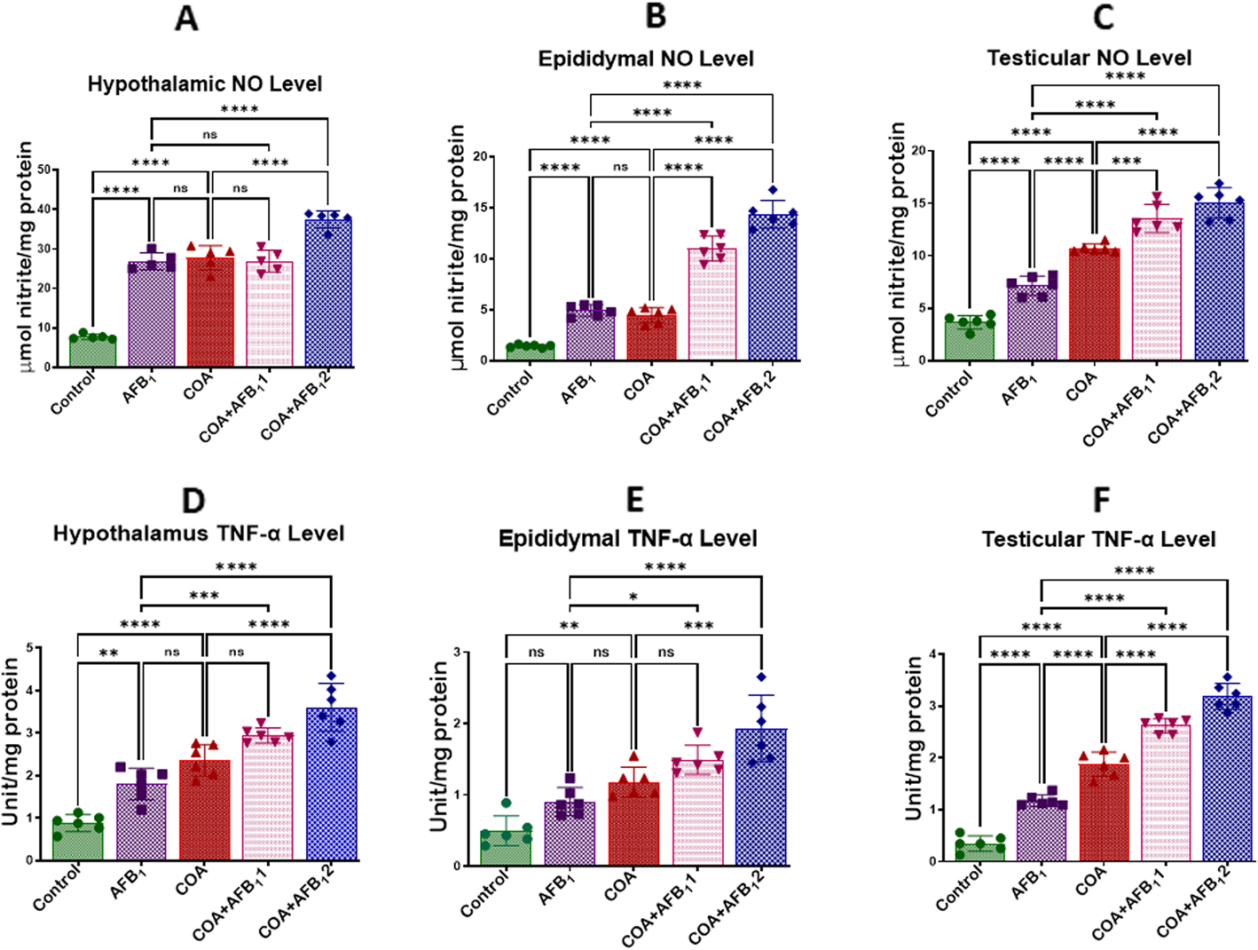
COA and AFB1 exposure Effect on the hypothalamic, epididymal and testicular levels of NO and TNF-α in male rats. COA, coartem at 5 mg/kg; AFB_1_1, aflatoxin at 35 µg/kg; AFB_1_2, aflatoxin at 70 µg/kg. The mean difference between the groups was compared with a one-way analysis of variance (ANOVA) followed by a post-hoc Bonferroni test. Bars represent the mean ± SD of 6 rats for NO and mean ± SD of 3 for TNF-α. Groups that differ significantly at *p* < 0.05,* p* < 0.01, *p* < 0.001* p* < 0.0001 are indicated by *, **, *** and ****, respectively; pairwise comparison lines represent compared groups. NO, nitric oxide; TNF-α, tumour necrotic factor.

The impact of co-exposure to COA and AFB1 on the inflammatory biomarkers in the hypothalamus, epididymis and testes of rats are displayed in Figs. 7 and 8A–F. Compared to the control, the animals treated with COA or AFB1 alone had significantly increased hypothalamic, epididymal, and testicular XO and MPO activities (Fig. 7A–F. A similar trend was observed in the hypothalamic, epididymal and testicular NO and TNF-α levels as groups treated with COA or AFB1 alone demonstrated a significant (*p* < *0.05*) increase in these markers when compared to the control animals (Fig. 8A–F). Additionally, as the concentration of AFB1 was increased, and that of COA was maintained, the tissue levels of XO, MPO, NO and TNF-α were significantly increased in the hypothalamus, epididymis, and testis compared to rat’s cohort treated with AFB1 alone (Figs. 7 and 8A–F). This indicates that exposure to AFB1 and COA can perturb the balance between pro-inflammatory/anti-inflammatory responses in favour of pro-inflammatory responses.

### Exposure to COA and AFB1 perturbs genomic stability in rats’ hypothalamus, testis, and epididymis

**Figure 9:**
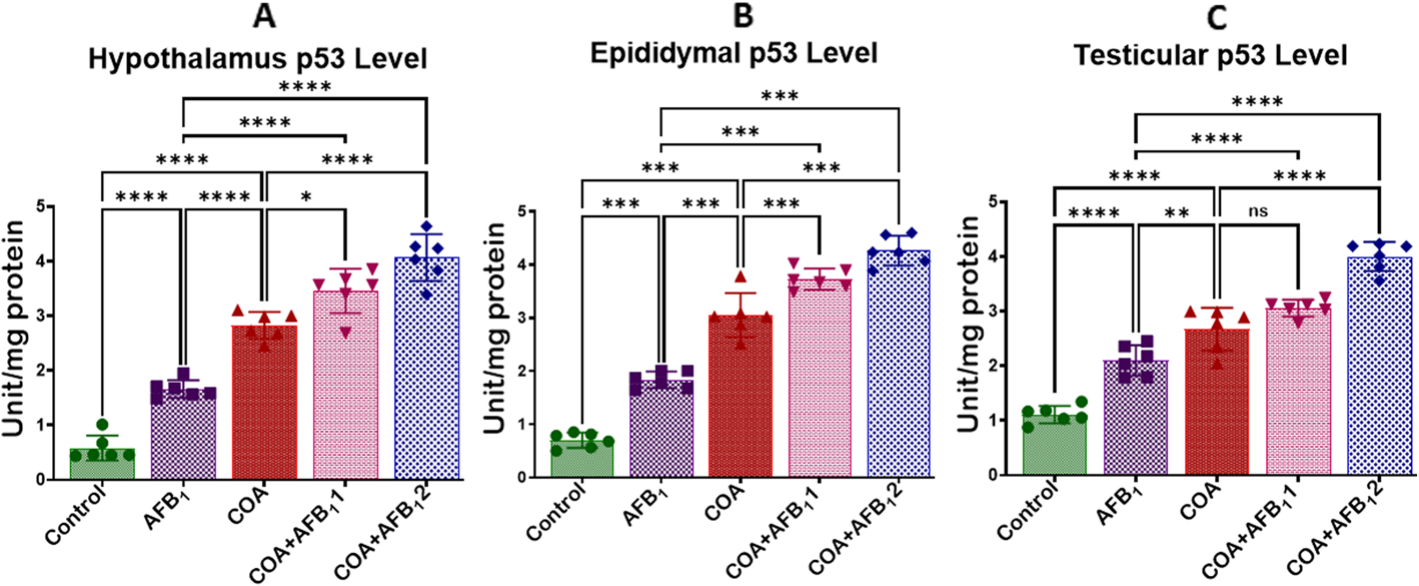
Effect of COA and AFB_1_ exposure on the hypothalamic, epididymal and testicular levels of TP53 in male rats. COA, coartem at 5 mg/kg; AFB_1_1, aflatoxin at 35 µg/kg; AFB_1_2, aflatoxin at 70 µg/kg. The mean difference between the groups was compared with a one-way analysis of variance (ANOVA) followed by a post-hoc Bonferroni test. Bars represent the mean ± SD of 3 rats. Groups that differ significantly at *p* < 0.05,* p* < 0.01, *p* < 0.001* p* < 0.0001 are indicated by *, **, *** and ****, respectively; pairwise comparison lines represent compared groups. TP53, tumour protein 53.

The impact of COA and AFB1 exposure on the level of p53 in the hypothalamus, epididymis and testes of rats is displayed in Fig. 9A–C. Compared to the control, the level of p53 increased significantly (*p* < *0.05*) in the hypothalamus, epididymis and testes of the animals treated with COA or AFB1. Further, co-exposure of animals to COA and AFB_1_ (35 and 70 µg/kg) exacerbated the condition as demonstrated by elevated levels of p53 in the hypothalamus, epididymis and testes of animals when compared to the animals that received COA or AFB_1_ alone. This indicates that COA and AFB1 co-exposure may rouse the genomic expression pattern of p53, a marker of genomic stability in cells exposed to toxic signals.

### Exposure to COA and AFB1 trigger regulated cell death in rats’ hypothalamus, testis, and epididymis

**Figure 10:**
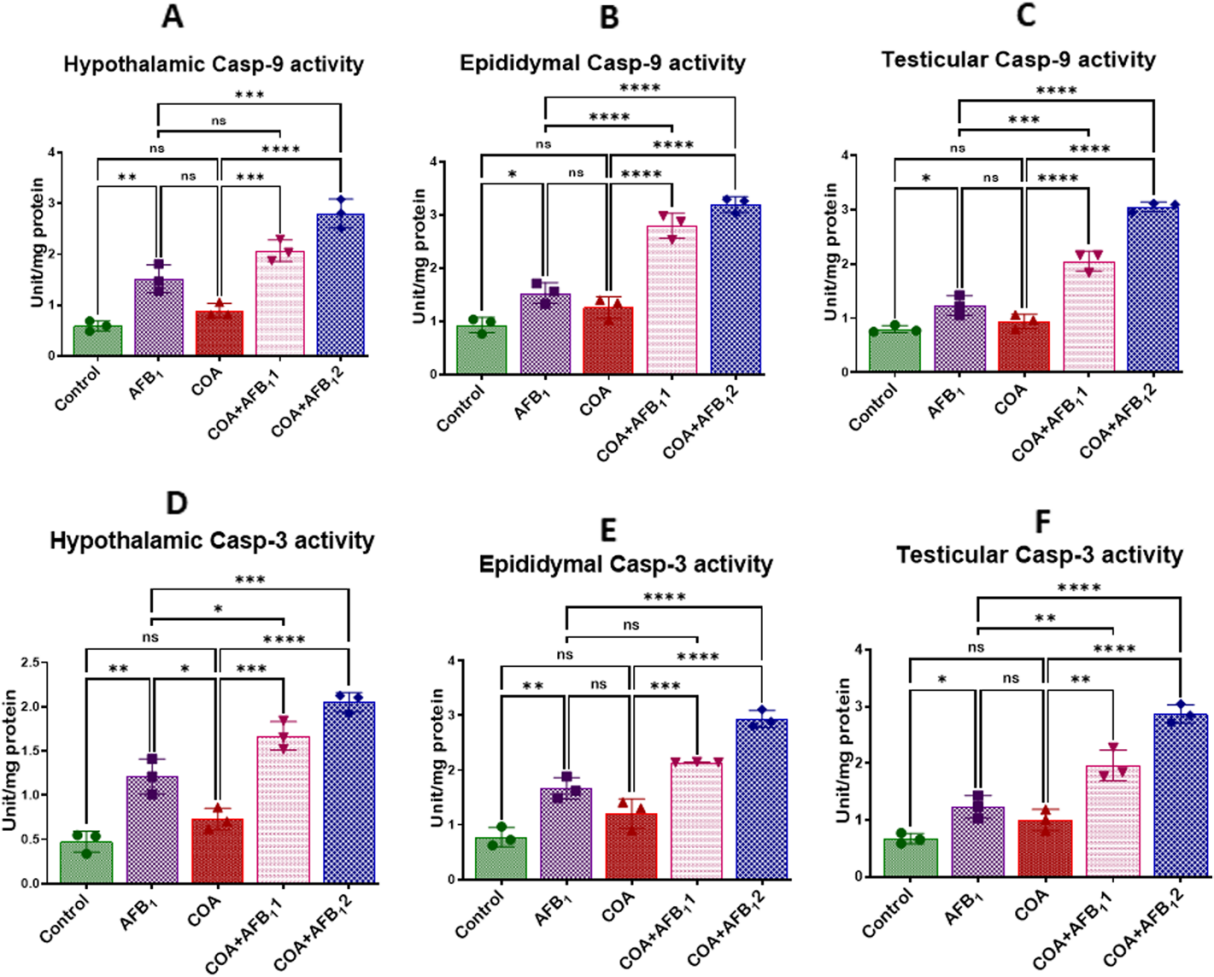
Effect of COA and AFB_1_ exposure on the hypothalamic, epididymal and testicular activities of caspase-9 and caspase-3 in male rats. COA, coartem at 5 mg/kg; AFB_1_1, aflatoxin at 35 µg/kg; AFB_1_2, aflatoxin at 70 µg/kg. The mean difference between the groups was compared with a one-way analysis of variance (ANOVA) followed by a post-hoc Bonferroni test. Bars represent the mean ± SD of 3 rats. Groups that differ significantly at *p* < 0.05,* p* < 0.01, *p* < 0.001* p* < 0.0001 are indicated by *, **, *** and ****, respectively; pairwise comparison lines represent compared groups. Casp, caspase.

The effects of combined exposure to COA and AFB1 on the activities of Casp-9 and Casp-3 in the hypothalamus, epididymis and testes of rats are displayed in Fig. 10A–F. Compared to the control, individual exposure to AFB1 significantly increased (*p* < *0.05*) the activities of Casp-9 and Casp-3 in the animals’ hypothalamus **(**Fig. 10A and D), epididymis (Fig. 10B and E), and testis (Fig. 10C and F). A non-significant increase (*p* > *0.05*) in the activities of the apoptotic proteins was observed in cohorts of rats exposed to only COA. Furthermore, co-exposure of animals to COA and AFB_1_ (35 and 70 µg/kg) exacerbated the condition, as demonstrated by a rapid increase in the activities of Casp-9 and Casp-3 in the hypothalamus, epididymis and testes of animals when compared to the animals that received COA or AFB_1_ alone. The findings indicate that co-exposure to COA and AFB1 may trigger regulated cell death in rats’ hypothalamus, testis, and epididymis.

### Exposure to COA and AFB1 impairs the histoarchitectural features of rats’ testis and epididymis

**Figure 11:**
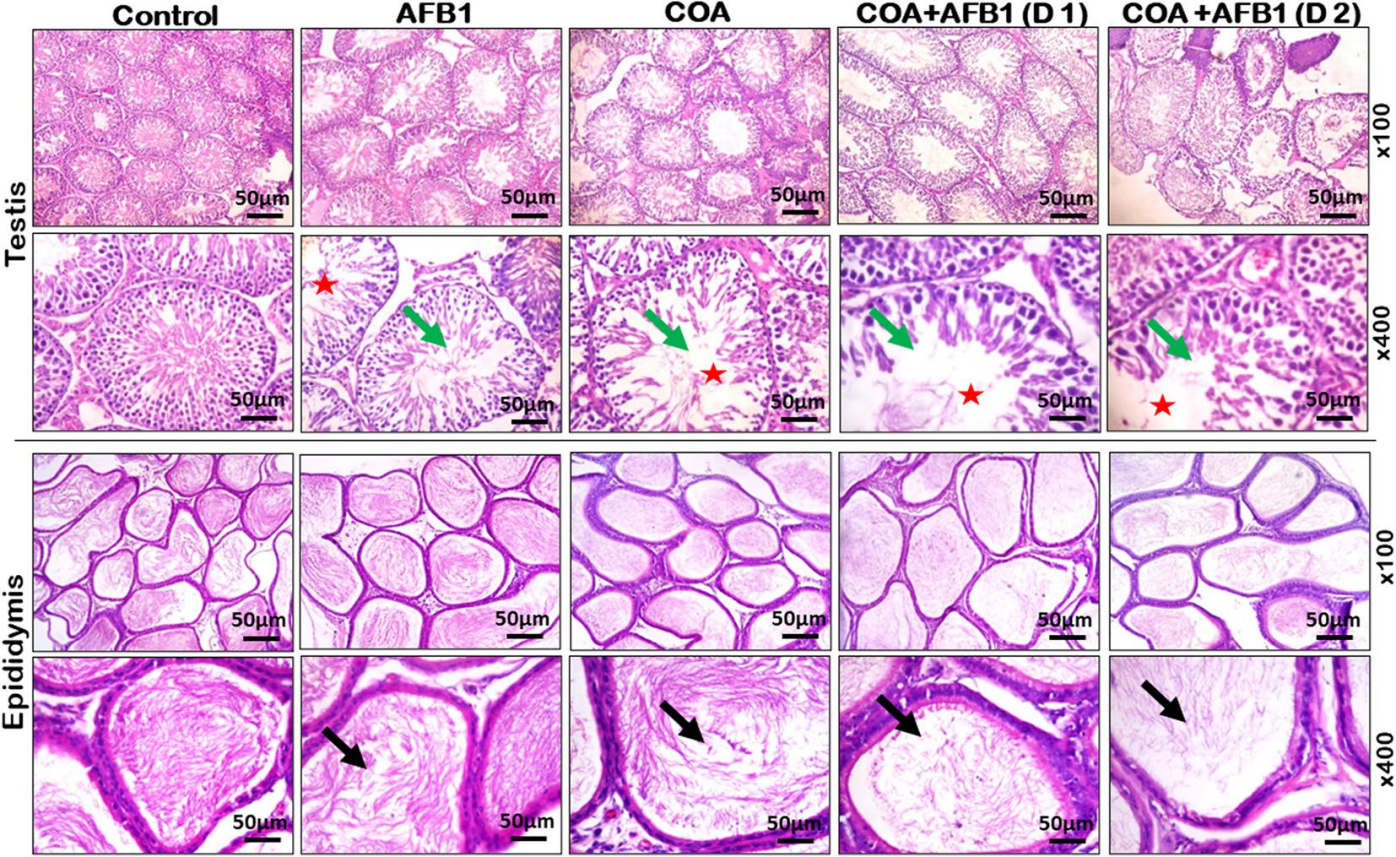
Representative photomicrographs of the testes and epididymis of experimental rats treated with Coartem (COA: mg/kg) and varying doses of aflatoxin B1 (AfB_1_ (D1): mg/kg) and (AfB_1_ (D2): mg/kg) for 28 consecutive days. The tissue sections were stained with Haematoxylin and Eosin, and the images were captured at two different magnifications (× 100 top row; × 400 bottom row). Control experimental rats show typical testicular and epididymal histology architecture, exemplified by distinct seminiferous tubules and abundant spermatozoa within the lumen: the Sertoli cells, spermatogonia and interstitial spaces presented with normal Leydig cells. There is a gradual but apparent regression in spermatozoa number in the lumen of experimental rats treated with COA alone. Similarly, co-treatment with COA and increasing doses of AfB_1_ (D1 and D2) increasingly impacted treated rats' testicular and epididymal tissue morphology with gradual interstitial erosion of the lumen (bold red stars). This observed erosion correlated with reduced sperm number in the epididymal lumen (black arrow), co-relating with increasing doses of AfB_1_ (doses 1 and 2) in rats co-treated with a constant amount of COA.

We further probe the impact of the exposure to COA and AFB1 on the histoarchitectural features of the testis and epididymis of rats, and the results are presented in Fig. 11. Compared to the rats in the control cohort, which show typical testicular and epididymal histology architecture, exemplified by distinct seminiferous tubules and abundant spermatozoa within the lumen, there is a gradual but apparent regression in spermatozoa number in the lumen of experimental rats treated with COA alone, as well as gradual interstitial erosion of the lumen in AFB1-treated groups. The results indicate that COA and AFB1 co-exposure might impair the architectural features of rats. The frequency of lesions and structural relevant aberrations semi-quantitatively identified in the tissue’s sections are depicted in Table 3.

**Table 3:** Histopathological aberration and lesion observed in experimental rats’ testis and epididymis treated with Aflatoxin B_1_ (AFB_1_) and Coartem™ (COA) for 28 consecutive days.

Parameters	Control	AFB_1_	COA	COA + AFB1_1_	COA + AFB1_2_
Testis					
Erosion of lumen	0	2	3	5	5
Degenerated tissue	0	2	2	0	4
Spermatozoa deficiency	0	1	3	4	4
Necrotic degeneration	0	0	1	1	1
Epididymis					
Erosion of sperm in the lumen	0	1	1	2	3
Tubular degeneration	0	1	1	2	2
Histoarchitecture	0	0	1	1	2

AFB1 alone: 0.070 mg/kg; COA alone: 5 mg/kg; AFB1_1_: 0.035 mg/kg; AFB1_2_: 0.07 mg/kg. Values are the number of rats with observed lesions in their tissues. Degree of lesion: 0–5; None (0); mild (1); mild-moderate (2); moderate (3); moderate to severe (4); and severe (5).

## Discussion

This study investigated the effects of co-exposure to a mycotoxin, AFB1 and the most familiar antimalarial drug in sub-Saharan Africa, COA, on the hypothalamic-pituitary-reproductive axis of male Wistar rats. The hypothalamus, the pituitary gland, and the accessory organs (testes and epididymis) make up the male reproductive axis, known as the hypothalamic-pituitary-gonadal axis (HPG axis). Our findings show that the integrity of experimental rats’ testis, epididymis and hypothalamus was adversely affected by co-exposure to COA and AFB1 (35 and 70 µg/kg).

The organosomatic indices of animals measure the toxic impact of xenobiotics on exposed animals^55^. Exposure of rats to COA or AFB_1_ alone led to a decrease in the body weight gain, organ weight and relative organ weight of the hypothalamus and epididymis of the animals compared to the control. However, the testicular and relative weights of the animals that received COA alone or COA and a low dose of AFB_1_ (35 µg/kg) were increased. Changes in mean body weight and the ratio of organ weight to body weight observed in the COA or AFB_1_ alone groups signal the beginning of tissue dysfunction and loss of body mass. The body weight gain, organ weight and relative organ weight of animals were not adversely affected by co-exposure to COA and AFB_1_. This could mean that co-treatment of male animals with COA and AFB_1_ has no significant effect on the body weight gain, organ weight and relative organ weight of male animals.

Furthermore, there was a noticeable reduction in sperm motility, epididymal sperm number and sperm viability in animals treated with COA or AFB_1_. This agrees with the study of Aprioku and Mankwe, who reported that treating animals with combined therapy of artemether and lumefantrine decreased sperm count, motility, and viability^56^. In addition, Owumi et al. have reported multiple studies on the ability of AFB_1_ to cause aberrations in sperm count, motility, and viability^6, 57, 58^. Simultaneously, there was an increase in the total sperm abnormalities of the rats co-exposed to COA and AFB_1_ (35 and 70 µg/kg). Aberrations in sperm quality and quantity may lead to infertility due to the sperm’s inability to enter the zonal pellucida and reach the site of fertilisation^59, 60^. These observations were aggravated in the rat cohorts treated with COA and AFB_1_. It can be argued that co-exposure of rats to COA and AFB1 (35 and 70 µg/kg) negatively impacts the quality and quantity of sperm, which could make the animals prone to infertility.

The significant biochemical alterations in the testes during spermatogenesis depend on the activities of some enzymes, including ACP, ALP G6PD and LDH. These enzymes are well-known markers of testicular metabolism and are frequently used to evaluate the survival of spermatogonial stem cells after toxicant exposure^61^. To produce and mature sperm, these enzymes must actively engage in the energy metabolism and stabilisation of the testes^61^. Acid phosphatases supply phosphate for the Sertoli cells and Leydig cells of the testis during development, growth, and maturation. A decline in the activity of ACP is a clinical sign of damage to the reproductive processes of the concerned animal^62^. LDH is an essential enzyme widely expressed in the testes and is known to control several processes critical for male fertility and sperm function^63^. To maintain the processes of meiosis, spermatogenesis, Sertoli cell maturation, and spermatogonial stem cells (SSCs) of the testis, the glycolytic pathway must be activated, and adenosine triphosphate (ATP) must be produced in anaerobic conditions^64^. In addition, to maintain spermatogenesis and steroidogenesis, G6PD in the hexose monophosphate shunt produces nicotinamide adenine dinucleotide phosphate (NADPH) and the sugar moiety, ribose 5-phosphate, which mitigates the energy requirement of round spermatids^64^.

Further, the hydroxylation of steroids required for spermatogenesis is carried out using NADPH produced by G6PD^65^. During spermatogenesis and testicular steroidogenesis, ALP, expressed in the Leydig cells and SSCs of the testis, controls several metabolic processes^66^. As demonstrated in the present study, there was a significant (*p* < *0.05*) decline in the activities of ACP, ALP, G6PD and LDH in the groups which received only COA or AFB1**.** This decline was further pronounced in the co-exposed groups. The decline in activities of testicular enzymes points to seminiferous epithelium degeneration and dysfunction of the hypothalamic-reproductive system. This study provides evidence that the ACP, ALP, G6PD, and LDH activities were inhibited by exposure to COA and AFB_1_ (35 and 70 µg/kg), which could impede spermatogenesis and steroidogenesis in the testes and, ultimately, poor reproductive health in male rats as affirmed by previous findings^26, 67^.

For healthy reproductive function, the HPG axis is a crucial system that controls spermatogenesis and reproductive hormone production^68^. The functioning of the male reproductive system depends on maintaining gonadotropins like LH and FSH, which regulate the hypothalamic-pituitary-testicular system. Typically, LH, FSH, and prolactin from the pituitary gland act on the testes’ Leydig, Sertoli, and germ cells to begin the production of testosterone and sperm^69^. Further, prolactin increases the Leydig cells’ LH receptors’ sensitivity to LH circulating in the blood, indicating their ability to increase testosterone levels and spermatogenesis^69^. With increased prolactin levels, LH and FSH hormones can be suppressed^68, 69^. When oxidative stress and inflammation are induced in the hypothalamic-pituitary-testicular system, prolactin is known to have a brief negative feedback loop effect on the expression of LH and FSH, thus suppressing the production of sperm cells and the activities of testicular enzymes^69^. Compared to the control animals of this study, the serum levels of testosterone, LH and FSH decreased.

In contrast, the level of prolactin was increased in the serum after exposure of animals to COA or AFB1 alone. This dysregulation was further exacerbated dose-dependent by co-exposure to COA and 35 and 70 µg/mg AFB1. Hyperprolactinemia has been associated with erectile dysfunction, most likely due to the reduction of testosterone synthesis by the action of prolactin on LH receptors^69, 70^. This observation demonstrates the toxic effects of co-exposure of experimental animals to COA and AFB_1_. The reduction in the serum levels of male reproductive hormones examined in this study indicates the ability of COA and AFB1 exposure to cause hormonal disruption and potentiate infertility.

The high rates of cell division by the germinal epithelium via spermatogenesis and metabolism by the Leydig cells, i.e. steroidogenesis, render the testes prone to increased oxygen consumption and, invariably, oxidative stress^71^. The testes have developed an array of both enzymatic and non-enzymatic antioxidants to protect themselves from oxidative damage and ensure their ability to produce potent sperm cells with the ability to cause fertilisation^71^. The epididymis provides a safe environment for the development and maturation of spermatozoa and protects them from oxidative stress-induced damage with endogenous antioxidants^72^. On the other hand, the hypothalamus maintains homeostasis in the body and can control every endocrine gland, including the reproductive hormones^73^; hence its role in regulating the reproductive function of animals is essential. Therefore, it is fortified with enzymatic and non-enzymatic antioxidants to aid the scavenging of free radicals. A defence system made up of the antioxidant enzymes SOD, CAT, GPx and GST and the non-enzymatic antioxidants GSH and TSH work to protect the cells of the male reproductive organs from oxidative damage while also maintaining normal testicular steroidogenesis, spermatogenic cell division, and epididymal epithelium effectiveness^74^. The rapid dismutation of superoxide anions (${\text{O}}_{{2}}^{ \bullet - }$) into hydrogen peroxide (H_2_O_2_) by SOD and the conversion of H_2_O_2_ into H_2_O by either CAT or GPx are the mechanisms by which antioxidant enzymes work^75^. GPx is more abundant in the testes^76^. This prevents ${\text{O}}_{{2}}^{ \bullet - }$ and H_2_O_2_ from participating in the Haber–Weiss and Fenton reactions to produce the highly toxic hydroxyl radical (OH•)^77^. The activity of GST, a complex group of enzymes that conjugate GSH, via the sulfhydryl group to electrophilic centres on a wide range of substrates in preparation for excretion from the cell is essential for the detoxification of peroxidised lipids and the metabolism of xenobiotics^71^. In this study, the groups treated with COA or AFB1 alone showed a significant decrease in SOD, CAT, GPx, GST antioxidant activities and the levels of GSH and TSH in the hypothalamus, epididymis and testis of the rats. This observed decrease in antioxidant activities was further exacerbated by co-exposure to COA and AFB1. The significant reduction in the activities and levels of these antioxidants renders the reproductive tissues of animals vulnerable to attacks by free radicals and peroxides, thus exposing these tissues to the toxic effects of COA and AFB1. This provides evidence that exposure of animals to COA and AFB1 has a debilitating impact on the HPG axis of the rats, which could damage the animals’ reproductive tissues and render them infertile.

Oxidative stress, which results when ROS production exceeds elimination by antioxidants, could be aggravated by exposure to xenobiotics and has been associated with male infertility^21, 78^. At physiological levels, RONS play an essential role in male fertility. ROS induces spermatozoa proliferation^79^, aids sperm motility and acrosome reaction, and functions as a cellular messenger in sperm cells^21^. However, the overproduction of ROS in the reproductive tissues of animals has pathological consequences on their fertility. ROS facilitate the removal of hydrogen from a fatty acid's hydrocarbon side chain, resulting in a carbon-centred lipid radical (L^•^) that reacts with oxygen to form a lipid peroxyl radical (LOO^•^), which can then respond with an additional fatty acid and initiate a chain reaction^80, 81^. Peroxidation of membrane lipids erodes the protective compounds of the cellular membrane and opens the cell up to damage^82^. In addition, oxidative stress in the hypothalamus can disrupt the release of hormones crucial to reproduction^83^.

The amount of RONS and malondialdehyde present in the examined organs measures the level of oxidative stress. Therefore, a significant increase in the levels of the oxidative stress biomarkers, specifically LPO and RONS, observed in the hypothalamus, epididymis and testis experimental animals treated with COA or AFB1 alone compared with the control indicates the ability of COA or AFB_1_ to increase the levels of RONS and LPO in the HPG axis of rats. Recent studies have confirmed that oxidative stress is the primary mechanism of AFB1-mediated testicular damage^84^. Excessive oxidative stress has been demonstrated to induce germ cell apoptosis in animals and impair the structural integrity of testes while also harming the spermatogenesis and steroidogenesis processes^85^. Findings from this study indicated that co-treatment with COA and AFB_1_ (35 and 70 µg/kg) exacerbated oxidative stress in the rats' hypothalamus, epididymis, and testis. This observation, coupled with the decrease in the antioxidant markers, indicates that the redox status of the examined organs has been compromised, thus leading to damage to the examined organs' tissue structure and increased ROS production in the experimental animals.

Inflammation is the host’s protective response to attack from microbes or tissue injury, which may be caused by the xenobiotics^86^. However, unresolved inflammation negatively correlates with spermatogenesis and steroidogenesis^87^. Inflammation has been found to decrease circulating hormone levels and prevent sperm development and motility, thus resulting in an agglutination^87^. ROS-induced inflammation can activate the MPO system of polymorphonuclear (PMN) cells and macrophages, producing even more ROS. XO catalyses the oxidation of hypoxanthine to uric acid and xanthine during purine metabolism with the generation of superoxide radicals as side products^88^. Uric acid produced from XO is a potent activator of the nuclear factor kappa-light-chain-enhancer of activated B cells (NFκB), which increases pro-inflammatory cytokines such as tumour necrotic factor alpha (TNF-α) and thus induction of inflammation^89^. It is well known that TNF-α controls cytokine production during an inflammatory response, which causes the cell to produce NO from the inducible nitric oxide (iNOS) synthase^90^.

NO is a physiologically and pathologically relevant compound in the male reproduction^91^. Physiologic levels of NO play significant roles in steroidogenesis, erection and penile functions, sperm capacitation, and acrosome reaction. It is also a regulator of cellular interactions in the testes^91^. However, pathologic conditions trigger the increased production of NO by upregulating the activities of the NO synthases^67^. Under pathologic conditions, NO acts as a pro-inflammatory mediator that induces oxidative stress, which has been shown to cause infertility^91^. Gonadal functions are disrupted by excessive NO production, which also causes oxidative damage to the germ cells and germ cell death^91^. It has also been shown that ROS generated by XO induce apoptosis of testicular cells^92^. In addition, LPO in spermatozoa may be worsened by the accumulation of XO activity by-products since spermatozoa cell membranes contain highly susceptible polyunsaturated fatty acids^93^. After depleting antioxidant defence mechanisms, increased cellular NO levels cause nitrosative stress, which modifies cellular lipids, proteins, and nucleic acids. In addition, the activation of MPO, an enzyme that produces hypochlorous acid and ROS, harms the reproductive tissues^94^. In the present study, the rat cohorts exposed to COA or AFB_1_ alone showed a significant increase in NO and TNF-α levels. COA or AFB_1_ alone also upregulated XO and MPO activities in the hypothalamus, epididymis and testis compared to the control. Co-exposure to these xenobiotics (COA and AFB_1_) exacerbated the increase in NO and TNF-α levels and XO and MPO activities in the examined organs. Elevated levels of pro-inflammatory cytokines in the reproductive organs are detrimental to spermatogenesis and have a reputation for causing male infertility^95, 96^.

One of the leading causes of poor sperm function is DNA damage, mainly caused by oxidative stress. It is a prevalent underlying cause of male infertility, repeated miscarriages, complex neuropsychiatric problems, and childhood malignancies in children whose fathers had faulty sperm cells. Oxidative stress harms sperm DNA, RNA transcripts, and telomeres^97^. Spermatozoa have a single, limited mechanism for detecting and repairing DNA damage, which makes them highly susceptible to oxidative stress^97^. The toxicity of AFB_1_ is ascribed mainly to its intermediate AFB1-Exo-8,9 epoxide (AFBO), a very unstable compound that reacts with cellular macromolecules, including deoxyribonucleic acid (DNA) and proteins^84^. p53 is a tumour suppressor protein activated in response to cellular stress, such as increased ROS production and DNA damage. Increased p53 levels have been correlated with exposure to AFB1^78, 98, 99^. Also, using the comet assay, artemisinin, the active agent in COA, is speculated to act by causing DNA damage in *Plasmodium falciparum*^100^ and cancer cells^101^. Therefore, the significantly higher levels of p53 observed in the hypothalamus, epididymis and testes of animals exposed to COA or AFB_1_ compared to the control indicates the DNA damage-causing effect of exposure to the drugs. p53 is increased to repair the DNA damage caused after exposure to the xenobiotics. The increased p53 is further worsened by co-exposure to the drugs, as seen in the animal cohorts treated with COA and AFB_1_ (35 and 70 µg/kg). To prevent damaged cells from progressing through the cell cycle, p53 is expressed to activate the DNA repair mechanisms of cells. However, prolonged exposure to DNA damage agents such as COA and AFB_1_ will result in the overexpression of p53, resulting in the arrest of the cell cycle and apoptosis of the cells. DNA damage and eventual overexpression of p53 in the testes will cause impaired spermatogenesis, negatively impacting the quality and quantity of sperm^102^.

The influx of pro-oxidants and pro-inflammatory mediators drives regulated cell death. Increased levels of these agents have been shown to induce genomic instability, which then triggers p53 upregulation—the guardian of the genome^103^. Expression of p53 will commit the cells to two fates through cell cycle arrest to ensure that the cues responsible for genomic instability are remediated or induce regulated cell death where such remediation is not feasible. As expected in the latter case, p53 induces the expression of p53-upregulated modulators of apoptosis (PUMA), which then perturbs the Bax/Bcl-2 ratio in favour of Bax (a pro-apoptotic protein)^104^. The translocation of Bax into the mitochondria of the representative tissues can orchestrate mitochondrial permeability transition pore and release cytochrome C into the cytoplasm, where it interacts with apoptotic peptidase activating factor 1 (Apaf-1) and procaspase-9 to form a complex known as apoptosome^8^. The formation of this complex is needed to convert procaspase-9 into caspase-9 (the initiator of apoptosis). Casp-9 then cleaves procaspase-3 into caspase-3 (the executioners of apoptosis). In this study, we found that AFB1 and COA orchestrated regulated cell death in rats' hypothalamus, epididymis, and testes by increasing caspase-9 and caspase-3. While we were unable to show the expression pattern of apoptotic proteins using western blot, we are confident that AFB1 and COA-mediated elevation in the levels of caspase-9 and caspase-3 as quantified by ELISA kits is ample evidence to support our claims that persistent exposure to AFB1 and COA could trigger ROS and RNS generations and production of pro-inflammatory molecules which can perturb genomic stability and commit the cell to apoptosis.

**Figure 12:**
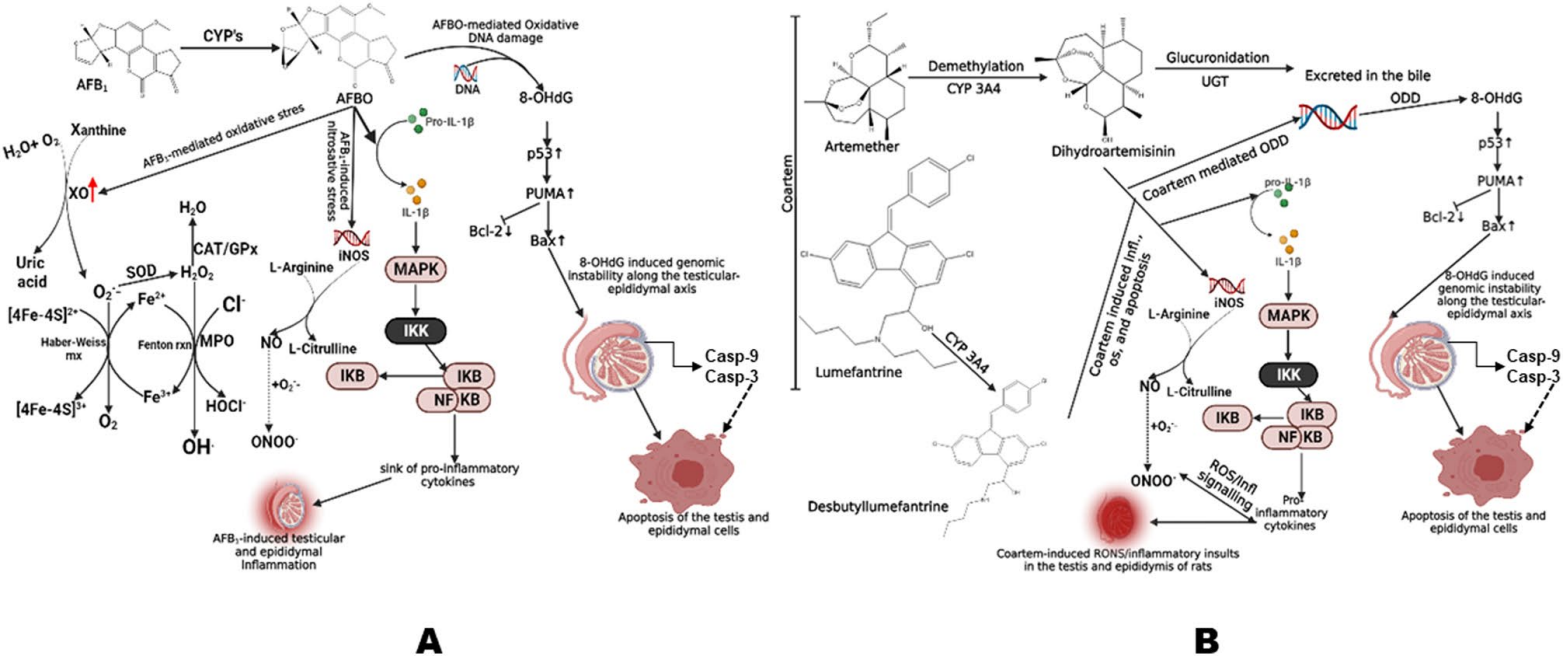
Proposed mechanisms of inadvertent exposure to dietary aflatoxin B_1_ (AfB_1_) and Coartem—artemether and lumefantrine, their biotransformation, mechanistic interaction and likely toxicity pathways in experimental rats treated for 28 consecutive days. (**A**) AFB_1_ is converted to AFBO by the CYP family of enzymes. AFBO, a potent intermediate, is immediately degraded by GST in the presence of GSH into aflatoxin B_1_ mercapturic acid, which is excreted in the urine. In unobstructed exposure to AFB_1_, AFBO accumulates in the hepatic and renal tissues and degrades xanthine into uric acid, with the simultaneous release of superoxide anion radical (O_2_^∙−^) and 8-OHdG (via oxidative DNA damage). Superoxide dismutase (SOD) mediates the dismutation of O_2_^∙−^ into H_2_O_2_. Excess H_2_O_2_ is then converted to molecular water by catalase (CAT) and glutathione peroxide (GPx). In the presence of chloride ion (Cl^−^), H_2_O_2_ is further converted to hypochloride (HOCl^−^) by myeloperoxidase (MPO). A ROS increase activates iNOS, which converts arginine to nitric oxide (NO). NO reacts with O_2_^∙−^ to form peroxynitrite (ONOO^−^). In the presence of Fe^2+^, H_2_O_2_ is converted to hydroxyl radical (^.^OH) (Fenton reaction). Excess ROS drives inflammation and apoptosis by acting on NF-kB and 8-OHdG, respectively. While activated, NF-kB mediates pro-inflammatory gene transcription, leading to inflammation. 8-OHdG upregulates the expression of p53, which upregulates the expression of PUMA. PUMA then activates Bax. Bax then enters the mitochondria and induces MPTP, releasing cytochrome C. Cytochrome C, combined with Apaf1 and procaspase-9, forms the apoptosome, converting procaspase-9 into activating Casp-9. Caspase-9 then activates procaspase-3 to active caspase-3, eventually executing programmed cell death. (**B**) Coartem comprises Artemether and lumefantrine. Following ingestion, Artemether is demethylated by CYP 3A4 into dihydroartemisinin (DHA), which is then conjugated with glucuronic acid by UDP-glucuronosyl transferase into an inactive intermediate that is excreted in the bile. At the same time, lumefantrine is acted upon by CYP 3A4 into desbutyllumefantrine. Accumulating DHA and desbutyllumefantrine will induce oxidative stress, inflammation, and DNA damage. The preceding changes will trigger the releases of a DNA adduct (8-OHdG), which mediate the upregulation of p53, PUMA and Bax and, subsequently, programmed cell death. Additionally, these potent intermediates can drive inflammation and oxidative stress by generating abundant ROS and RNS as well as pro-inflammatory mediators—all plunging the hepatic and renal tissues into prolonged inflammation. Coartem, artemether and lumefantrine; AFB1, aflatoxin B1; AFBO, aflatoxin B_1_-8, 9 epoxides; CYP, cytochrome P450; ROS, reactive oxygen species; RNS, reactive nitrogen species; NF-kB, nuclear factor kappa B; TNF-α: MPTP, mitochondrial permeability transition pore; Bcl-2, B-cell lymphoma 2; Bax, Bcl-2 associated X; Apaf1, apoptotic peptidase activating factor 1^20^. They were created by Arunsi Uche Okuu using BioRender (Created with BioRender.com).

Histoarchitectural assessment of the target tissues is essential to validate the experimental data elucidated in this study. Our results showed that cohorts of rats treated with AFB1 and COA showed atypical testicular and epididymal histology features exemplified by apparent regression of spermatozoa number and interstitial erosion of the lumen. As a result of testicular and epididymal atrophies, some functional testicular enzymes such as ALP, ACP, G6PD, and LDH are released from the lumen into the cytosol, resulting in reduced testicular levels of the enzymes, and impaired spermatogenesis and steroidogenesis—an indication of poor reproductive function^26, 67^. Moreso, the sustained generation of ROS/RNS and pro-inflammatory molecules could mediate the recruitment and trafficking of inflammatory cells into the testis and epididymis^105^, where they compromise the pro-inflammatory/anti-inflammatory cytokine balance and induced chronic inflammation as well as necrosis^106, 107^ if the guardian of the genome (p53) fails to induce DNA repair enzymes and resolve inflammatory responses. Taken together, we modelled that the probable mechanisms for the observed COA and AFB1-mediated toxicities might be through the activation of the activities of cytochrome P450 enzymes such as CYP1A2, CYP 2C9, CYP 2C19, CYP2D6 and 3A4, NF-κB, IRFs, TLRs, Caspase-9, and Caspase-3 as well as the downregulation of the expression of p53, Nrf-2, NQO1, HO-1, Bcl-2 (Fig. 12) in the hypothalamus, testis, epididymis of rats.

## Conclusions

Overall, we have demonstrated that co-exposure of rats to COA or AFB1 resulted in oxidative stress, inflammation, genomic instability, and apoptosis in rats' hypothalamus, testis, and epididymis, thereby disrupting the spermatogenic process and maturation of the male reproductive organs of the experimental rats. Similar perturbations have been observed in other experimental rats' organ systems^54, 108^. Since chronic COA abuse and inadvertent exposure to AFB1 are becoming important public health issues, especially in Sub-Sahara Africa, it is pertinent to revalidate these chemicals that might induce male infertility in animals and humans if allowed to accumulate in the tissues long-term.

## Data Availability

The datasets used and analysed during the current study are available from the corresponding author upon reasonable request.
